# Polycaprolactone Electrospun Scaffolds Produce an Enrichment of Lung Cancer Stem Cells in Sensitive and Resistant EGFRm Lung Adenocarcinoma

**DOI:** 10.3390/cancers13215320

**Published:** 2021-10-22

**Authors:** Emma Polonio-Alcalá, Marc Rabionet, Santiago Ruiz-Martínez, Sònia Palomeras, Rut Porta, Carmen Vásquez-Dongo, Joaquim Bosch-Barrera, Teresa Puig, Joaquim Ciurana

**Affiliations:** 1 Product, Process and Production Engineering Research Group (GREP), Department of Mechanical Engineering and Industrial Construction, University of Girona, 17003 Girona, Spain; emma.polonio@udg.edu (E.P.-A.); m.rabionet@udg.edu (M.R.); 2 New Therapeutic Targets Laboratory (TargetsLab)-Oncology Unit, Department of Medical Sciences, Faculty of Medicine, University of Girona, 17003 Girona, Spain; santiago.ruiz@udg.edu (S.R.-M.); sonia.palomeras@udg.edu (S.P.); rporta@iconcologia.net (R.P.); cavasquez.girona.ics@gencat.cat (C.V.-D.); 3 Medical Oncology Department, Catalan Institute of Oncology, 17007 Girona, Spain; jbosch@iconcologia.net; 4 Department of Pathology, Dr. Josep Trueta University Hospital, 17007 Girona, Spain

**Keywords:** NSCLC, cancer stem cells, 3D cell culture, electrospinning, CD133, Vimentin

## Abstract

**Simple Summary:**

The culture of lung cancer stem cells (LCSCs) is not possible using traditional flat polystyrene surfaces. The study of these tumor-initiating cells is fundamental due to their key role in the resistance to anticancer therapies, tumor recurrence, and metastasis. Hence, we evaluated the use of polycaprolactone electrospun (PCL-ES) scaffolds for culturing LCSC population in sensitive and resistant EGFR-mutated lung adenocarcinoma models. Our findings revealed that both cell models seeded on PCL-ES structures showed a higher drug resistance, enhanced levels of several genes and proteins related to epithelial-to-mesenchymal process, stemness, and surface markers, and the activation of the Hedgehog pathway. We also determined that the non-expression of CD133 was associated with a low degree of histological differentiation, disease progression, distant metastasis, and worse overall survival in EGFR-mutated non-small cell lung cancer patients. Therefore, we confirmed PCL-ES scaffolds as a suitable three-dimensional cell culture model for the study of LCSC niche.

**Abstract:**

The establishment of a three-dimensional (3D) cell culture model for lung cancer stem cells (LCSCs) is needed because the study of these stem cells is unable to be done using flat surfaces. The study of LCSCs is fundamental due to their key role in drug resistance, tumor recurrence, and metastasis. Hence, the purpose of this work is the evaluation of polycaprolactone electrospun (PCL-ES) scaffolds for culturing LCSCs in sensitive and resistant EGFR-mutated (EGFRm) lung adenocarcinoma cell models. We performed a thermal, physical, and biological characterization of 10% and 15%-PCL-ES structures. Several genes and proteins associated with LCSC features were analyzed by RT-qPCR and Western blot. Vimentin and CD133 tumor expression were evaluated in samples from 36 patients with EGFRm non-small cell lung cancer through immunohistochemistry. Our findings revealed that PC9 and PC9-GR3 models cultured on PCL-ES scaffolds showed higher resistance to osimertinib, upregulation of ABCB1, Vimentin, Snail, Twist, Sox2, Oct-4, and CD166, downregulation of E-cadherin and CD133, and the activation of Hedgehog pathway. Additionally, we determined that the non-expression of CD133 was significantly associated with a low degree of histological differentiation, disease progression, and distant metastasis. To sum up, we confirmed PCL-ES scaffolds as a suitable 3D cell culture model for the study of the LCSC niche.

## 1. Introduction

Lung cancer is the leading cause of cancer-related mortality worldwide among men and women [1]. The 5-year survival rate is 19.4%, and about 57% of lung cancer cases are diagnosed at advanced stages of the disease when surgical resection is not possible and radio- and chemotherapy show a response rate of roughly 25% [2,3]. Non-small cell lung cancer (NSCLC) is the most common subtype, and approximately 40% of cases are diagnosed as adenocarcinoma [4]. The discovery of activating mutations in the tyrosine kinase domain of the epidermal growth factor receptor (EGFR) led to the development of different targeted therapies, such as gefitinib or osimertinib. Despite the good initial response to these therapies, most patients develop progressive disease, acquiring resistance through different mechanisms [5,6]. Therefore, there is an indubitable need to better understand the disease in order to identify new biomarkers. 

Cancer stem cells (CSCs) are a small subpopulation within the tumor responsible for cancer recurrence, metastasis, and resistance to current therapies. These tumor-initiating cells have self-renewal and pluripotency capacities [7,8,9]. The stemness potential is closely regulated by several transcription factors, such as Sox2, Oct-4, and Nanog [10,11,12]. Consequently, lung cancer stem cells (LCSCs) play a key role in the occurrence and development of lung cancer by driving intratumor heterogeneity [13]. Different surface markers have been linked to this malignant subpopulation, such as CD133, CD166, CD24, or CD90 [14,15,16,17]. Cancer cells are also capable of removing cell–cell and cell–matrix interactions to migrate from the primary tumor to other organs through the epithelial-to-mesenchymal transition (EMT) process [18]. EMT is also related to cancer stemness and resistance to anticancer therapies [19]. Furthermore, researchers have reported that the canonical Wnt/β-catenin and the Hedgehog signaling pathways are crucial for the LCSC population [20,21].

Lung cancer is traditionally studied using two-dimensional (2D) cell culture and animal models. Nonetheless, these methodologies have some limitations. Monolayer culture does not fully mimic the tumor microenvironment where the extracellular matrix (ECM) has an essential role in some processes, for example gene expression and drug response. At the same time, cancer cells affect ECM deposition, degradation, and remodeling, influencing tumor progression and invasiveness [22]. Although 2D cell culture is a well-established, simple, and economical method, flat surfaces alter apical-basal polarity, nutrient and oxygen distribution, soluble gradients, and cell proliferation, morphology, and interactions [23]. Additionally, monolayer culture causes the differentiation of CSCs, which lose their stemness behavior [24]. Animal models offer a natural in vivo environment, but they are expensive and their use is discouraged due to the 3R (Replacement, Reduction, and Refinement) principle. Only 8% of drugs tested in animal systems remain in clinical trials; hence, they do not predict human responses properly [25]. Thus, more reliable and suitable in vitro culture systems are required to reduce the number of animals for scientific research. 

In the past few years, researchers have studied several three-dimensional (3D) strategies to simulate physiological lung tissue conditions, such as decellularized lung scaffolds [26], hydrogel matrices [27], tumors-on-chip [28], patient-derived organoids [29], cell-line spheroids [30], or biopolymeric structures [31,32], among others. Chen et al. highlighted that not only oncogenic changes but also the surrounding microenvironment are crucial for the definitive cell phenotype in lung adenocarcinoma [33]. Cell proliferation, genetic and protein expression, and response to drugs are modified in cells cultured on 3D models compared to monolayer culture [22]. Besides this, researchers have pointed out that mechanical signals induced by cell-generated physical force cause changes in cell morphology, adhesion, alignment, differentiation, and migration [34]. Electrospinning is a cost-effective and simple technique that produces nanofibers similar to the ECM structure [35]. Moreover, electrospun (ES) scaffolds provide a 3D configuration that possesses a high surface area-to-volume ratio, allowing cell attachment. Polycaprolactone (PCL) is a synthetic polymer frequently selected for scaffold manufacturing. It is commonly used with electrospinning technology because of its low melting temperature (around 60 °C) and easy adjustability. This low-cost material is suitable for biomedical and cell culture applications due to its biocompatibility, bioresorbability, and long-term biodegradability [36]. Previously, our research group reported that PCL-ES platforms increase the CSC population in breast cancer [37].

The main aim of this study was to evaluate the effectiveness of PCL-ES scaffolds for culturing the LCSC niche. Sensitive and resistant EGFR-mutated (EGFRm) lung adenocarcinoma cell models were seeded on the resulting meshes from two different PCL concentrations (10 and 15% *w*/*v*), and then several LCSC features were analyzed. Moreover, Vimentin and CD133 tumor expression were also evaluated in 36 samples from patients with EGFRm NSCLC in order to validate in vitro results.

## 2. Materials and Methods

### 2.1. Chemicals & Reagents

Polycaprolactone (PCL, Mn 80,000 g/mol), paraformaldehyde, Triton^TM^ X-100, 3-(4,5-dimethyl-2-thiazolyl)-2,5-diphenyl-2H-tetrazolium bromide (MTT), bovine serum albumin (BSA) (≥98.0%), phenylmethylsulfonylfluoride (PMSF), TWEEN^®^ 20, and primers (Table S1) were purchased from Sigma-Aldrich (St. Louis, MO, USA). BSA Fraction V pH for Western blotting (min. 96%), acetone (min. ≥99.8%), and tris-buffered saline (TBS) were provided from PanReac AppliChem (Gatersleben, Germany). Ethanol absolute was obtained from Labkem-Labbox Labware S.L. (Barcelona, Spain) and Qiazol was acquired by Qiagen (Hilden, Germany). Roswell Park Memorial Institute (RPMI-1640) medium, penicillin/streptomycin 10,000 U/mL, phosphate-buffered saline (PBS), and trypsin 10× were supplied from Lonza (Basilea, Switzerland) and fetal bovine serum (FBS) was purchased from HyClone (Logan, UT, USA). DC Protein Assay, 40% acrylamide solution, and Clarity^TM^ Western ECL Substrate were obtained from Bio-Rad (Hercules, CA, USA). Rhodamine-phalloidin was acquired from Cytoskeleton Inc. (Denver, CO, USA) and 4,6-diamidino-2-phenylindole (DAPI) was provided from BD Pharmingen (Franklin Lakes, NJ, USA). GeneJET RNA Purification Kit, nitrocellulose membranes, and West Femto Maximum Sensitivity Substrate were supplied by Thermo Fisher Scientific Inc. (Waltham, MA, USA). High Capacity cDNA Archive Kit was purchased from Applied Biosystems (Foster City, CA, USA) and qPCRBIO SyGreen Mix Lo-Rox was obtained from PCR Biosystems Inc. (Wayne, PA, USA). Lithium dodecyl sulfate (LDS) sample buffer and sample reducing agent were acquired from Invitrogen (Carlsbad, CA, USA). Lysis buffer was purchased from Cell Signaling Technology (CST) Inc. (Danvers, MA, USA). Antibodies (Table S2) were provided from CST, Abcam (Cambridge, UK), ProteinTech^®^ (Manchester, UK), and Roche Diagnostics (Basilea, Switzerland). Osimertinib was kindly supplied by AstraZeneca (Cambridge, UK). UltraView Universal DAB Detection Kit and Amplification Kit were purchased from Roche Diagnostics.

### 2.2. Electrospun Scaffolds Manufacturing

PCL was dissolved in acetone at 10% and 15% (*w*/*v*) under agitation at 60 °C for 24 h. Scaffolds were manufactured by an electrospinning machine (Spraybase, Dublin, Ireland). The solution was moved to a 20 mL syringe connected to a stainless steel 18G needle (inner diameter of 0.8 mm) through a polytetrafluoroethylene tube (inner diameter of 1 mm). The distance between the emitter and collector was 10 cm. The electrospinning process was established by Syringe Pump Pro software (New Era Pump Systems; Farmingdale, NY, USA). For the 10% PCL concentration, 8.5 mL of solution was ejected at a voltage of 9 kV and a flow rate of 6 mL/h. For the 15% PCL concentration, 5 mL was ejected at a voltage of 7 kV and a flow rate of 6 mL/h. The resulting meshes were cut and sterilized as described elsewhere [38].

### 2.3. Thermal Analysis

#### 2.3.1. Thermogravimetric Analysis

The thermal stability of PCL-ES scaffolds was determined by thermogravimetric analysis (TGA) using Mettler-Toledo TGA/DSC 1 (Mettler-Toledo; Columbus, OH, USA). Samples (11.45 mg for 10% PCL; 10.03 mg for 15% PCL) were performed at a heating rate of 10 °C/min at a temperature range from 30 to 700 °C under a nitrogen atmosphere. 

#### 2.3.2. Differential Scanning Calorimetry

The calorimetric behavior of PCL-ES structures was measured by differential scanning calorimetry (DSC) using TA Instruments Q2000 (TA Instruments; New Castle, DE, USA). Samples (4.71 mg for 10% PCL; 5.42 mg for 15% PCL) were contained in an aluminum pan under a dynamic nitrogen atmosphere (50 mL/min) at a heating rate of 10 °C/min from 30 to 100 °C. 

#### 2.3.3. Dynamic Mechanical Analysis

The viscoelastic property of PCL-ES scaffolds was measured by dynamic mechanical analysis (DMA) using Mettler-Toledo DMA/SDTA861e (Mettler-Toledo). The size samples were 5.50 mm of length, 7.00 mm of width, and 0.65 mm of thickness. DMA was performed in tensile test mode, at a heating rate of 5 °C from −85 °C to 45 °C with 1 Hz of frequency and 50 μm of amplitude. 

### 2.4. PCL Electrospun Scaffolds Observation through Scanning Electrone Microscopy

The weight and thickness of PCL-ES scaffolds were measured by the Sartorius ED224S analytical balance (Sartorius; Göttingen, Germany) and the Mahr Micromar 40 EWV digital micrometer (Mahr; Göttingen, Germany), respectively. Samples were cut and stuck with a carbon bioadhesive. To provide conductivity, a layer of colloidal silver surrounded the sample and afterwards, the carbon was eliminated by the Quorum Emitech K950 Turbo Evaporator (Emitech, Kent, UK). The microarchitecture was examined by the Hitachi S4100 field emission scanning electron microscope (FESEM) (Hitachi; Tokyo, Japan) and images were captured by Quartz PCi software (Quartz; Vancouver, Canada). The average values of fiber diameter, surface porosity, and pore area were determined from top and bottom sides via ImageJ software (National Institutes of Health; Bethesda, MD, USA).

### 2.5. Weight Degradation Assay

PCL-ES structures were firstly weighed using the analytical balance (Sartorius). Afterward, they were sterilized, relocated to non-adherent cell culture 12-well plates (Sarstedt, Nümbrecht, Germany), and 2 mL of supplemented medium was added into the wells. Scaffolds were maintained in the incubator for 3, 6, 14, or 28 days, and then they were washed twice with PBS, air-dried, and weighed again. Control samples were directly air-dried and weighed after their sterilization.

### 2.6. Protein Adsorption Assay

Sterilized scaffolds were soaked in 2 mL of supplemented medium and blank samples in PBS. Structures were placed in the incubator at 37 °C and 5% CO_2_ for 3 and 6 days. In order to guarantee that only proteins attached to meshes were analyzed, PCL-ES scaffolds were put into new wells after their PBS rinsing. The protein amount from each sample was calculated through a BSA standard curve by DC Protein Assay. Absorbance was measured at 700 nm in a microplate reader (Bio-Rad).

### 2.7. Cell Models

Human EGFRm lung adenocarcinoma PC9 and its gefitinib and osimertinib resistant derivative PC9-GR3 models were kindly provided by Dr. R. Rosell and Dr. M. A. Molina (Barcelona, Spain). Cells were routinely grown in RPMI-1640 medium supplemented with 10% FBS, and 50 U/mL penicillin/streptomycin. Cells were maintained at 37 °C and 5% CO_2_ atmosphere, regularly monitored, and remained mycoplasma-free.

### 2.8. Three-Dimensional Cell Culture

Sterilized scaffolds were put in non-adherent cell culture plates (Sarstedt) and immersed in supplemented medium for 30 min at 37 °C and in a 5% CO_2_ atmosphere to ensure cell attachment. The appropriate cell density was prepared in 50 μL of medium for 12-well scaffolds and 75 μL for 6-well ones. PC9 and PC9-GR3 models were seeded in scaffolds as previously described [39].

### 2.9. Nucleus and Cytoplasm Elongation

Cells were seeded on adherent coverslips (Sarstedt) and sterilized scaffolds for 3 and 6 days. After the PBS rinsing, cells were fixed with 4% (*w*/*v*) paraformaldehyde, permeated using 0.2% (*v*/*v*) Triton X-100, blocked using 3% (*w*/*v*) BSA, and dyed the actin cytoskeleton by rhodamine-phalloidin (1:250) and the nucleus by DAPI (1:1000). Fluorescence was examined through a Nikon A1R confocal laser scanning microscope (CLSM) (Nikon, Tokyo, Japan) and all images were taken by Nikon NIS-Elements AR v4.10 software (Nikon). Image J (National Institutes of Health, Bethesda, MD, USA) software was used to determine nuclear and cytoplasmic elongation. As described elsewhere [40], five cells from each image were randomly chosen to measure the length and width of the nucleus and cytoplasm.

### 2.10. Cell Viability Assays

To compare cell viability rate in 2D and 3D cell cultures, sensitive and resistant models were cultured on adherent cell culture 12-well plates and 10–15% PCL-ES scaffolds for 3 and 6 days. Afterward, PCL-ES structures were rinsed twice with PBS and placed in new wells. Finally, the MTT assay was performed as previously described [38].

To investigate osimertinib resistance of cells cultured on 2D and 3D culture, PC9 and PC9-GR3 models were seeded as previously mentioned. After 3 or 6 days, cells were treated with different concentrations of osimertinib for 48 h. Thereafter, the MTT assay was performed.

### 2.11. Quantitative Real-Time PCR Analysis

Cells cultured on 2D and PCL-ES scaffolds for 3 and 6 days were trypsinized, recollected, and resuspended in 700 μL of Qiazol. In order to obtain RNA from samples, the GeneJET RNA Purification Kit was performed following the manufacturer’s protocol and the RNA isolated was quantified by a NanoDrop 2000 Spectophotometer (ThermoFisher Scientific). RNA was reverse-transcribed into complementary DNA (cDNA) using the High Capacity cDNA Archive Kit. Gene expression levels were detected using primers (Table S1) and qPCRBIO SyGreen Mix Lo-Rox through the QuantStudio3 Real-Time PCR System (ThermoFisher Scientific Inc., Waltham, MA, USA). Results obtained were transformed using the standard formula 2^ΔCT^ and normalized to the housekeeping GAPDH.

### 2.12. Immunoblotting Analysis 

Cells cultured on 2D and PCL-ES scaffolds for 3 and 6 days were trypsinized, recollected and lysed by vortexing every 5 min for 30 min in ice-cold lysis buffer with 100 μg/mL PMSF. Protein concentration was calculated through a BSA standard curve by DC Protein Assay. Equal amounts of protein were heated in LDS and reducing agent for 10 min at 70 °C. Thereafter, total protein was separated by SDS-polyacrylamide gel (SDS-PAGE) and moved to nitrocellulose membranes. Membranes were incubated for 3 h at room temperature in blocking buffer (5% BSA in TBS 0.05% Tween (TBS-T)) and overnight at 4 °C with the corresponding primary antibody (Table S2) diluted in blocking buffer. Specific horseradish peroxidase (HRP)-conjugated secondary antibodies were incubated for 90 min at room temperature before being detected in the Bio-Rad ChemiDoc^TM^ MP Imaging System (Bio-Rad Laboratories, Inc., Hercules, CA, USA) using a chemiluminescent HRP substrate Clarity^TM^ Western ECL Substrate or West Femto Maximum Sensitivity Substrate.

### 2.13. Selection of Patients

Between 2006 and 2019, at the Dr. Josep Trueta University Hospital (Girona, Spain), 45 patients met inclusion criteria. Clinical characteristics of the patients and pathological features of the tumors were analyzed retrospectively and obtained from the medical records. However, only 36 patients had sufficient tumor sample for immunohistochemical analysis. The samples were from biopsy, but all patients had received EGFR-tyrosine kinase inhibitors (TKIs) at some point.

### 2.14. Immunohistochemistry Assay of Tissue Samples 

CD133 and Vimentin tumor expression were determined by immunohistochemistry (IHC) in 3 µm thick slides from formalin-fixed paraffin-embedded tissue blocks of the primary tumor using a BenchMark ULTRA-Ventana instrument (Roche Diagnostics). The UltraView Universal DAB Detection Kit and the Amplification Kit were used for the detection of CD133 and Vimentin antibodies. A negative control was included by using mouse IgG at a comparable concentration instead of the primary antibody. Kidney and colon tissue were also examined as a control expression of CD133 and Vimentin, respectively. CD133 expression was considered as positive when at least 1% of the cells showed cytoplasmic and/or membrane staining. Vimentin expression was classified as positive when 10% or more of the cells were stained.

### 2.15. Data Analysis 

The statistical analysis was performed through the IBM SPSS software (Version 25.0; SPSS Inc., Chicago, IL, USA) and R software (Version 4.0.4; The R Foundation, Vienna, Austria). For in vitro experiments, they were performed at least three times and the results obtained are expressed as mean ± standard error of the mean (SEM). For samples from patients, categorical variables are summarized as counts and percentages, and continuous variables as the number of non-missing observations, the mean ± standard deviation (SD), or the median and interquartile range [IQR], depending on the distribution of the variable. Categorical variables were compared by Fisher’s exact tests. Overall Survival (OS) and Progression Free Survival (PFS) probabilities were estimated according to the method of Kaplan–Meier. Statistical differences between curves were calculated using the log-rank test. For two-group comparisons, parametric data were analyzed by Student’s *t* test and non-parametric data by Mann–Whitney U test. For more than two groups’ comparisons, parametric data were analyzed by one-way analysis of variance (ANOVA) using Bonferroni or Tamhane’s T2 post hoc test and non-parametric data were analyzed by Kruskal–Wallis test. Levels of significance were established at *p* < 0.050 and are represented by asterisks, as follows: *p* < 0.050 (*), *p* < 0.010 (**), and *p* < 0.001 (***). 

## 3. Results

### 3.1. Characterization of PCL-ES Scaffolds

#### 3.1.1. Thermal Characterization of PCL-ES Scaffolds

The thermal stability of structures was tested by TGA (Figure S1a,b). The first stage of the curve, from 30 °C to 380 °C with a slight weight loss of about 10%, corresponded to water vaporization and the elimination of unstable fragments. The weight degradation occurred from 387 °C to 430 °C for 10%-PCL-ES meshes and from 388 °C to 429 °C for 15%-PCL ones. After their decomposition, 10% and 15%-PCL-ES supports showed a residual weight of 9.88% and 9.66%, respectively.

The thermal properties of scaffolds were revealed by DSC (Figure S1c,d). A single peak was observed in both 3D platforms corresponding to melting temperature (T_m_) which was 59.66 °C for 10%-PCL-ES matrices and 59.52 °C for 15%-PCL ones. Regarding the enthalpy of fusion (ΔH_f_), 10% and 15%-PCL-ES meshes absorbed 74.90 J/g and 73.69 J/g, respectively.

The structural and viscoelastic behavior of 3D supports were examined by DMA (Figure S1e,f). We determined a storage modulus (E’) value of 6.25 MPa and 7.22 MPa at 25 °C (room temperature), and 4.52 MPa and 5.40 MPa at 37 °C (physiological conditions) for 10% and 15%-PCL-ES platforms, respectively. In terms of the Tan Delta curve, the glass transition temperature (T_g_) was −42.56 °C and −49.65 °C for 10% and 15%-PCL-ES structures, respectively.

#### 3.1.2. Microarchitecture of PCL-ES Scaffolds

The top (TS) and bottom (BS) sides of 3D meshes were displayed using SEM (Table 1) to expose their microarchitecture and measure their filament diameter, porosity, and pore area. 

One filament population was confirmed in 10%-PCL-ES matrices, whereas 15%-PCL ones exhibited two subpopulations of filaments (Figure S2). Additionally, beads (non-filamentous polymer) were observed in 10%-PCL-ES platforms. On the other hand, 15%-PCL-ES scaffolds showed higher fiber diameter, lower surface porosity, and larger pore area compared to 10%-PCL ones, in a significant way (Table 2). Significant differences were also found between top and bottom sides in the fiber diameter (329.69 ± 33.97 nm for TS, 297.94 ± 24.89 nm for BS; *p =* 0.007) and the surface porosity (66.13 ± 1.29% for TS, 70.34 ± 1.20% for BS; *p =* 0.036) of 10%-PCL-ES structures and the fiber diameter (1501.42 ± 570.10 nm for TS, 1979.60 ± 376.64 nm for BS; *p =* 1. 000 × 10^−4^) and pore area (0.70 ± 0.35 μm^2^ for TS, 0.76 ± 0.36 μm^2^ for BS; *p =* 0.019) of 15%-PCL ones.

#### 3.1.3. Effect of the Sterilization Process and Medium Immersion on PCL-ES Scaffolds

**Figure 1:**
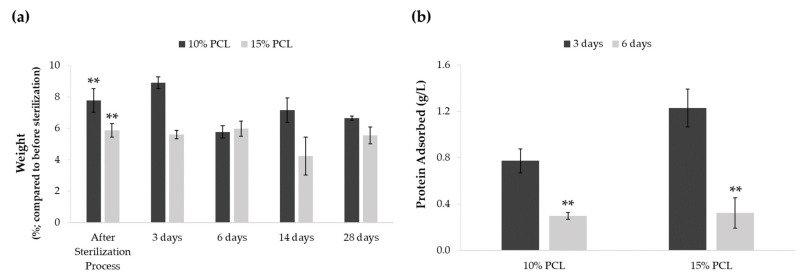
(**a**) Effect of sterilization process and medium soaking on the weight of PCL-ES scaffolds for 3, 6, 14, and 28 days. Results are shown as mean ± SEM from at least three independent experiments. Levels of statistical significance are indicated as ** (*p* > 0.010) compared to the weight before the sterilization process. (**b**) Capacity to adsorb protein from medium of PCL-ES scaffolds after 3 and 6 days of incubation. The results are shown as mean ± SEM from at least three independent experiments. Levels of statistical significance are indicated as ** (*p* > 0.010) compared to 3 days of incubation.

A weight degradation assay was performed to discern whether the sterilization procedure and medium soaking altered PCL-ES matrices (Figure 1a). The process of sterilization resulted in an increase of approximately 5–7% of their weight. Significant differences were found between the weight before and after sterilization in 3D supports. However, no significant variations were found between their weight after sterilization and medium immersion for 3, 6, 14, or 28 days. The medium soaking neither changed the weight of PCL-ES platforms throughout the 28-day period. 

We also investigated the scaffold capacity to adsorb protein from the medium on their surface after 3 and 6 days of incubation (Figure 1b). Both 3D structures were able to adsorb protein, which was significantly greater after 3 days than 6 days. Moreover, 15%-PCL-ES meshes exhibited a higher capacity to adsorb protein than 10%-PCL ones after 3 days.

### 3.2. Morphology of Sensitive and Resistant EGFRm Lung Adenocarcinoma Cell Models Cultured on PCL-ES Scaffolds

**Figure 2:**
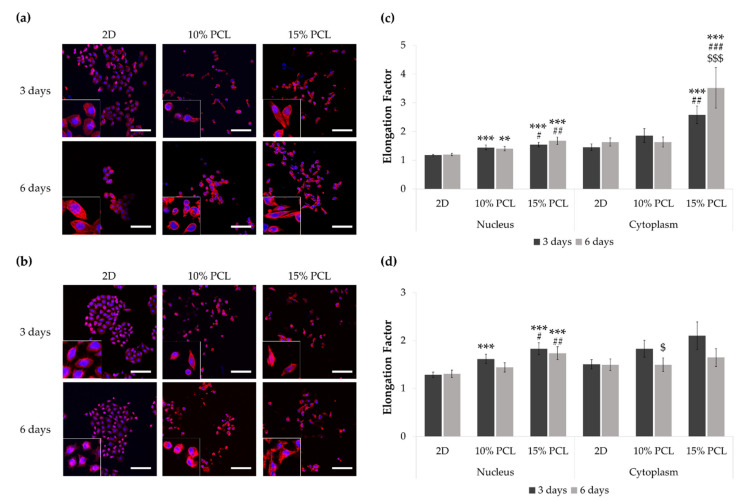
Images of (**a**) PC9 and (**b**) PC9-GR3 cell models cultured on monolayer, 10% and 15%-PCL-ES scaffolds for 3 and 6 days displayed by a confocal laser scanning microscope (CLSM) at a magnification of ×200 (scale bars: 100 µm) and partial pictures enlarged (×3). The actin cytoskeleton was stained with rhodamine-phalloidin (red) and the nucleus with DAPI (blue). Nuclear and cytoplasmic elongation factors from (**c**) PC9 and (**d**) PC9-GR3 cell models cultured on monolayer, 10% and 15%-PCL-ES scaffolds. Levels of statistical significance are indicated as *, #, $ (*p* < 0.050), **, ## (*p* < 0.010), and ***, ###, $$$ (*p* < 0.001). The symbol * indicates the comparison with monolayer, $ indicates the comparison with 3 days of culture, and # indicates the comparison with 10%-PCL-ES scaffolds.

To examine possible changes in cell morphology, PC9 and PC9-GR3 models were cultured on 2D and 3D (10% and 15%-PCL-ES) matrices for 3 and 6 days. The stained nucleus and actin cytoskeleton were displayed using CLSM (Figure 2a,b).

PC9 cells seeded on 3D platforms showed a significantly higher nucleus elongation compared to the monolayer and 10%-PCL ones (Figure 2c). Additionally, a significantly larger cytoplasmic lengthening was observed on cells grown on 15%-PCL-ES scaffolds for 3 and 6 days. Regarding culture time, PC9 cultured on 15%-PCL-ES structures also exhibited a more extended cytoplasm for 6 days than 3 days.

PC9-GR3 seeded on 15%-PCL-ES meshes for 3 and 6 days showed a significantly larger nucleus extension in comparison with 2D and 10%-PCL ones (Figure 2d). After 3 days, cells grown on 10%-PCL-ES supports also demonstrated a significantly higher elongated nucleus in contrast to the monolayer. It was observed a tendency to elongate the cytoplasm in cells seeded on 3D culture for 3 days in contrast to 2D. Nonetheless, PC9-GR3 grown on 10%-PCL-ES scaffolds for 6 days exhibited a shrunken cytoplasm compared to those grown for 3 days. The largest elongation of nucleus and cytoplasm were determined in cells seeded on 15%-PCL-ES meshes compared to the monolayer, for 6 days in PC9 and 3 days in PC9-GR3.

**Figure 3:**
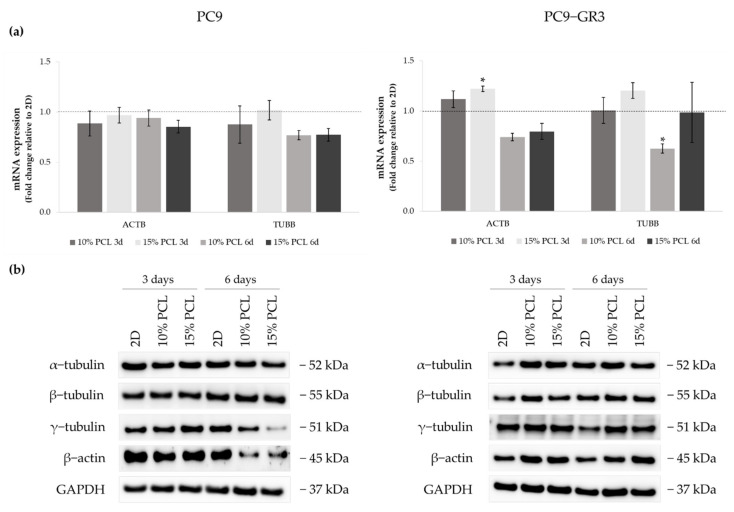
(**a**) *ACTB* and *TUBB* mRNA levels of PC9 and PC9-GR3 cell models cultured on monolayer, 10% and 15%-PCL-ES scaffolds for 3 and 6 days. mRNA expression was normalized against the GAPDH gene. All cell culture conditions were compared to 2D, which was normalized to 1 (marked by the dotted line) and shown as fold change. The results are shown as mean ± SEM from at least three independent experiments. Levels of statistical significance are indicated as * (*p* < 0.050) compared to 2D. (**b**) α-tubulin, β-tubulin, γ-tubulin, and β-actin protein expression of PC9 and PC9-GR3 models cultured on monolayer, 10% and 15%-PCL-ES scaffolds for 3 and 6 days. The 2D culture was used as an internal control and GAPDH as a loading control. The results shown are representative from at least three independent experiments.

Actin and tubulin were analyzed by RT-qPCR and Western blot (Figure 3) in order to clarify whether cells changed their expression as a consequence of 3D culture. The uncropped immunoblottings can be found in Figure S3. 

Although no changes were observed in *ACTB* expression in PC9, β-actin protein levels were decreased in cells cultured on 3D supports for 6 days. *TUBB* mRNA expression and γ-tubulin protein levels were also diminished in the same culture conditions. No alterations were detected in α- and β-tubulin protein levels. 

Regarding the PC9-GR3 cell model, *ACTB* mRNA levels were upregulated in cells cultured on 3D platforms for 3 days compared to 2D, being statistically significant in 15%-PCL ones. β-actin protein expression was also increased in cells seeded on 3D culture for 3 and 6 days, despite the *ACTB* reduction in scaffolds for 6 days. Cells grown on 3D structures exhibited an increase of α- and β-tubulin proteins expression for 3 days and an increase of γ-tubulin for 6 days. No changes were exhibited in *TUBB* mRNA levels, except for the significant reduction shown in 10%-PCL-ES meshes compared to the monolayer for 6 days.

### 3.3. Viability of Sensitive and Resistant EGFRm Lung Adenocarcinoma Cell Models Cultured on PCL-ES Scaffolds

**Figure 4:**
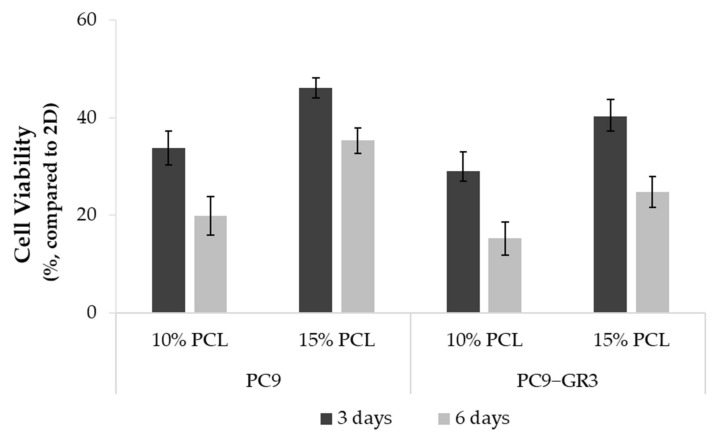
Cell viability of PC9 and PC9-GR3 cell models cultured on monolayer, 10% and 15%-PCL-ES scaffolds for 3 and 6 days. The results are shown as mean ± SEM from at least three independent experiments. All cell culture conditions were compared to 2D, which was normalized to 100%.

Differences in the viability of PC9 and PC9-GR3 cell models cultured on PCL-ES matrices or monolayer were studied through the MTT assay for 3 and 6 days (Figure 4). In both models, cells grown on 3D culture exhibited a lower rate compared to 2D. Cells seeded on 15%-PCL-ES meshes showed a higher viability than on 10%-PCL ones. Furthermore, it was observed that there was a tendency to decrease cell viability in cells cultured on 3D supports after 6 days in contrast to 3 days.

### 3.4. Evaluation of EGFR Status in Sensitive and Resistant EGFRm Lung Adenocarcinoma Cell Models Cultured on PCL-ES Scaffolds

**Figure 5:**
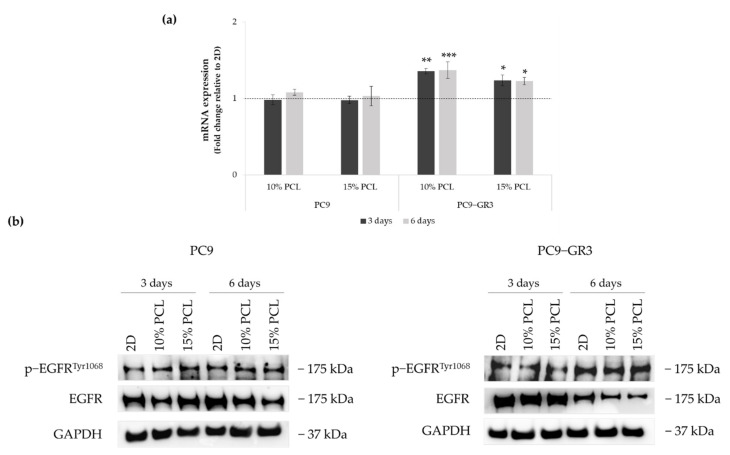
(**a**) *EGFR* mRNA levels of PC9 and PC9-GR3 cell models cultured on monolayer, 10% and 15%-PCL-ES scaffolds for 3 and 6 days. mRNA expression was normalized against GAPDH gene. All cell culture conditions were compared to 2D, which was normalized to 1 (marked by the dotted line) and shown as fold change. Results are shown as mean ± SEM from at least three independent experiments. Levels of statistical significance are indicated as * (*p* < 0.050), ** (*p* < 0.010), and *** (*p* < 0.001) compared to 2D. (**b**) EGFR protein expression of PC9 and PC9-GR3 models cultured on monolayer, 10% and 15%-PCL-ES scaffolds for 3 and 6 days. The 2D culture was used as an internal control and GAPDH as a loading control. The results shown are representative from at least three independent experiments.

The status of EGFR in PC9 and PC9-GR3 cell models cultured on PCL-ES scaffolds was evaluated after 3 and 6 days of culture (Figure 5). The uncropped immunoblottings can be found in Figure S3.

Although no changes were observed in *EGFR* mRNA expression and phosphorylated EGFR protein levels in PC9 seeded on 3D matrices, a slight reduction in total EGFR protein expression was observed in 10%-PCL-ES meshes after 3 days of culture and in both 3D platforms after 6 days.

*EGFR* mRNA levels were significantly higher in PC9-GR3 grown on PCL-ES structures. However, total EGFR protein expression was reduced in 3D supports after 6 days of culture. No changes were exhibited in phosphorylated EGFR expression.

### 3.5. Study of LCSC population in Sensitive and Resistant EGFRm Lung Adenocarcinoma Cell Models Cultured on PCL-ES Scaffolds

#### 3.5.1. Resistance to Osimertinib of Sensitive and Resistant EGFRm Lung Adenocarcinoma Cell Models Cultured on PCL-ES Scaffolds

To evaluate the capacity of PCL-ES matrices to culture the LCSC population, the resistance to osimertinib was investigated in PC9 and PC9-GR3 cell models seeded on 2D or 3D culture for 3 or 6 days, and then treated with the EGFR-TKI for an extra 48 h.

**Figure 6:**
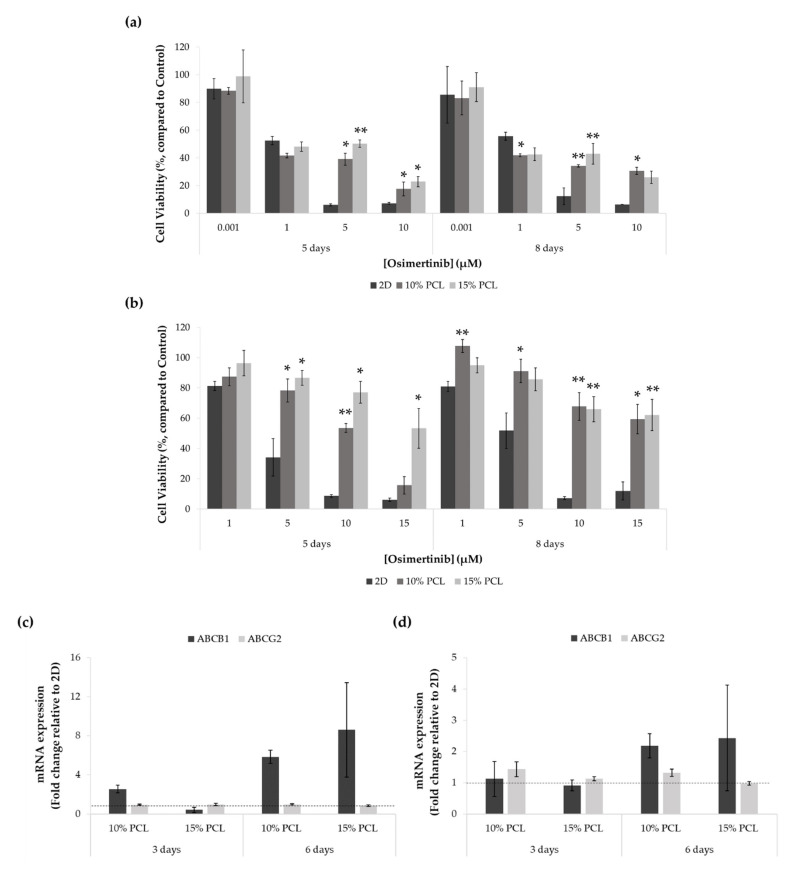
Cell viability of (**a**) PC9 and (**b**) PC9-GR3 cell models cultured on monolayer, 10% and 15%-PCL-ES scaffolds for 3 and 6 days and then treated with osimertinib for 48 h. Results are expressed as the percentage of surviving cells (mean ± SEM) compared to control (untreated cells) from at least three independent experiments. Levels of statistical significance are indicated as * (*p* < 0.050) and ** (*p* < 0.010) compared to 2D. *ABCB1* and *ABCG2* mRNA levels of (**c**) PC9 and (**d**) PC9-GR3 models cultured on monolayer, 10% and 15%-PCL-ES scaffolds for 3 and 6 days. mRNA expression was normalized against the GAPDH gene. All cell culture conditions were compared to 2D, which was normalized to 1 (marked by the dotted line) and shown as fold change. The results are shown as mean ± SEM from at least three independent experiments.

As shown in Figure 6a, no differences were found between PC9 seeded on PCL-ES structures and 2D after the treatment with 0.001 and 1 μM of osimertinib. Moreover, cells grown on 10%-PCL-ES meshes for 6 days exhibited significantly lower cell viability in comparison with control. Nevertheless, at the highest concentrations of osimertinib, PC9 cultured on 3D supports was significantly more resistant than on 2D culture.

Regarding the PC9-GR3 model, cells seeded on 3D culture were more resistant to osimertinib compared to the monolayer in all treatments assayed, as displayed in Figure 6b. As the EGFR-TKI concentration increased, the differences exhibited between 2D and 3D culture became more evident. 

*ABCB1* and *ABCG2* mRNA expression was also determined through RT-qPCR in both cell models cultured on PCL-ES platforms for 3 and 6 days (Figure 6c,d).

*ABCB1* levels were increased in cells cultured on 10%-PCL-ES structures for 3 days in PC9 and both 3D meshes after 6 days in PC9 and PC9-GR3 models. No changes were found in *ABCG2* expression in PC9 in any cell culture condition. However, *ABCG2* was slightly higher in PC9-GR3 seeded on 10%-PCL-ES meshes for 3 and 6 days.

#### 3.5.2. Epithelial-to-Mesenchymal Transition (EMT) of Sensitive and Resistant EGFRm Lung Adenocarcinoma Cell Models Cultured on PCL-ES Scaffolds

**Figure 7:**
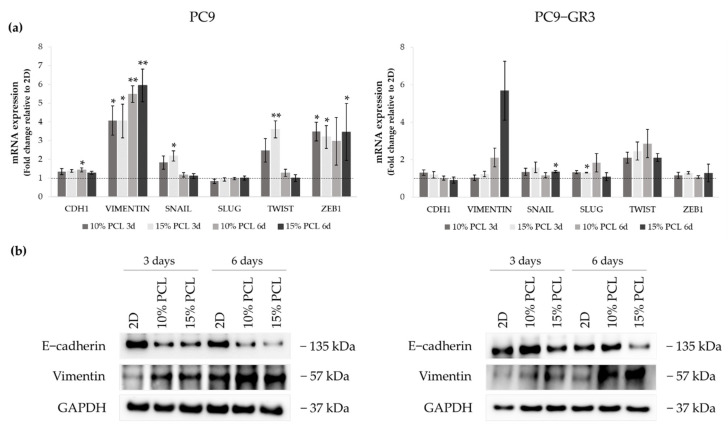
(**a**) *CDH1*, *VIMENTIN*, *SNAIL*, *SLUG*, *TWIST*, and *ZEB1* mRNA levels of PC9 and PC9-GR3 cell models cultured on monolayer, 10% and 15%-PCL-ES scaffolds for 3 and 6 days. mRNA expression was normalized against the GAPDH gene. All cell culture conditions were compared to 2D, which was normalized to 1 (marked by the dotted line) and shown as fold change. The results are shown as mean ± SEM from at least three independent experiments. Levels of statistical significance are indicated as * (*p* < 0.050) and ** (*p* < 0.010) compared to 2D. (**b**) E-cadherin and Vimentin protein expression of PC9 and PC9-GR3 models cultured on monolayer, 10% and 15%-PCL-ES scaffolds for 3 and 6 days. The 2D culture was used as an internal control and GAPDH as a loading control. The results shown are representative from at least three independent experiments.

We examined different transcription factors that trigger EMT, such as Snail, Slug, Twist, and Zeb1, by RT-qPCR, and E-cadherin and Vimentin by RT-qPCR and immunoblotting to determine the capacity of PCL-ES scaffolds to induce this process (Figure 7). The uncropped Western blots can be found in Figure S4 and Figure S5.

*CDH1* mRNA expression was slightly increased in PC9 grown on PCL-ES supports, being statistically significant in 10%-PCL ones compared to 2D after 6 days of culture. Nonetheless, E-cadherin protein levels were clearly diminished in cells on 3D culture after 3 and 6 days. *VIMENTIN* mRNA expression was significantly higher in PC9 cultured on 3D for 3 and 6 days in comparison with the monolayer, which is in agreement with its protein levels. Regarding the transcriptional factors, a significant enhancement of *SNAIL* and *TWIST* was shown in PC9 cultured on 15%-PCL-ES scaffolds for 3 days. No changes were found in *SLUG*. *ZEB1* mRNA levels were approximately three times greater in cells seeded on 3D in contrast to 2D, being statistically significant for both PCL-ES matrices after 3 days and only 15%-PCL ones after 6 days of culture. 

Although no changes in *CDH1* were observed in PC9-GR3, a reduction in E-cadherin protein levels was determined in cells grown on 15%-PCL-ES meshes for 6 days. mRNA and protein expression of Vimentin were higher in 3D supports after 6 days of culture. *SNAIL* and *SLUG* expression were significantly increased in PC9-GR3 cultured on 15%-PCL-ES platforms compared to the monolayer after 6 days and 3 days of culture, respectively. *TWIST* mRNA levels were approximately two times larger in cells seeded on 3D in comparison with 2D, but no changes were found for *ZEB1*.

#### 3.5.3. Self-Renewal, Stemness and Pluripotency Markers of Sensitive and Resistant EGFRm Lung Adenocarcinoma Cell Models Cultured on PCL-ES Scaffolds

**Figure 8:**
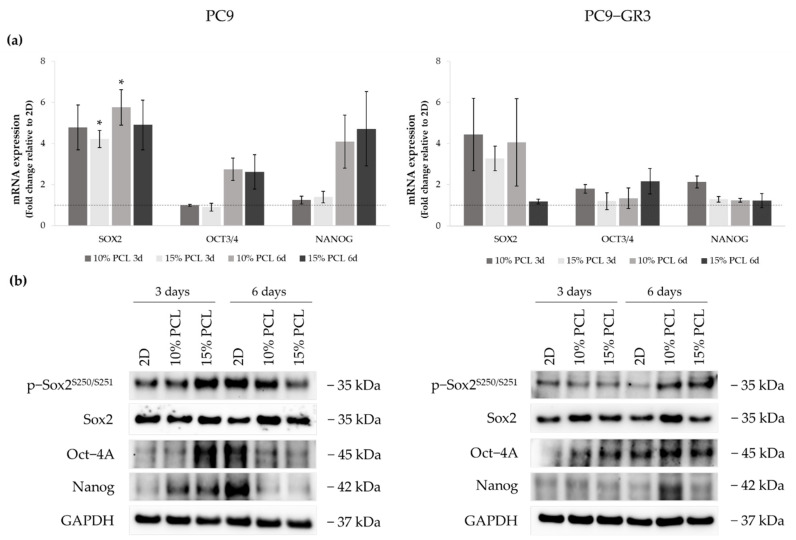
(**a**) *SOX2*, *OCT3/4*, and *NANOG* mRNA levels of PC9 and PC9-GR3 cell models cultured on monolayer, 10% and 15%-PCL-ES scaffolds for 3 and 6 days. mRNA expression was normalized against the GAPDH gene. All cell culture conditions were compared to 2D, which was normalized to 1 (marked by the dotted line) and shown as fold change. The results are shown as mean ± SEM from at least three independent experiments. Levels of statistical significance are indicated as * (*p* < 0.050) compared to 2D. (**b**) Sox2, Oct-4A, and Nanog protein expression of PC9 and PC9-GR3 models cultured on monolayer, 10% and 15%-PCL-ES scaffolds for 3 and 6 days. The 2D culture was used as an internal control and GAPDH as a loading control. The results shown are representative from at least three independent experiments.

Sox2, Oct-4, and Nanog expression were determined in 3D culture by RT-qPCR and Western blot to examine the capacity of PCL-ES scaffolds to culture this malignant subpopulation (Figure 8). The uncropped immunoblottings can be found in Figure S4 and Figure S5.

*SOX2* mRNA levels increased about five times in PC9 grown on PCL-ES matrices, being statistically significant in 15%-PCL ones after 3 days and 10%-PCL ones after 6 days of culture in contrast to the monolayer. Sox2 total protein levels were slightly greater in 3D after 6 days of culture. *OCT3/4* and *NANOG* expression were also larger in cells cultured on 3D for 6 days compared to 2D. Nevertheless, phosphorylated Sox2 and total Oct-4A protein were enhanced on cells seeded on 15%-PCL-ES structures for 3 days, and Nanog in both 3D meshes in comparison with the monolayer, but then their levels were diminished after 6 days of culture.

PC9-GR3 cultured on 10%-PCL-ES supports for 3 days caused a slight enhancement of *SOX2*, *OCT3/4*, and *NANOG* mRNA expression in contrast to 2D. An important increase of *SOX2* was also shown in cells grown on 10%-PCL-ES platforms for 6 days and on 15%-PCL ones after 3 days of culture. Phosphorylated levels of Sox2 were higher in 3D culture after 6 days of culture, but Oct-4A protein levels were larger after 3 days compared to the monolayer. Although no changes were observed in mRNA expression, PC9-GR3 seeded on 10%-PCL-ES structures for 6 days produced a slight increase in Oct-4A and Nanog protein levels.

#### 3.5.4. Membrane Receptors of Sensitive and Resistant EGFRm Lung Adenocarcinoma Cell Models Cultured on PCL-ES Scaffolds

**Figure 9:**
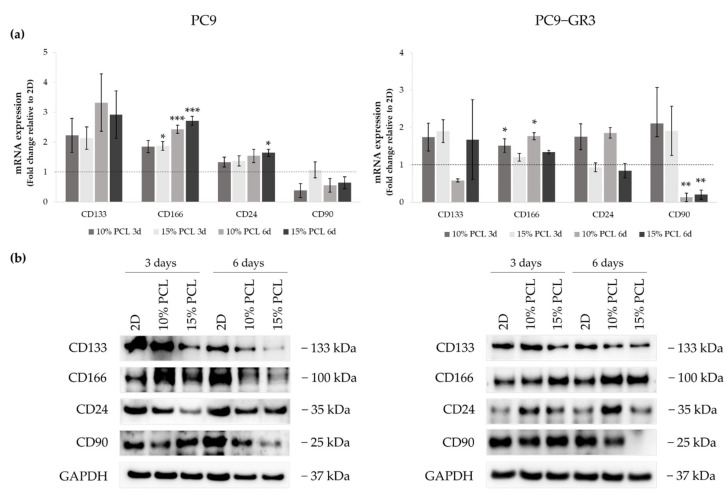
(**a**) *CD133*, *CD166*, *CD24*, and *CD90* mRNA levels of PC9 and PC9-GR3 cell models cultured on monolayer, 10% and 15%-PCL-ES scaffolds for 3 and 6 days. mRNA expression was normalized against the GAPDH gene. All cell culture conditions were compared to 2D, which was normalized to 1 (marked by the dotted line) and shown as fold change. The results are shown as mean ± SEM from at least three independent experiments. Levels of statistical significance are indicated as * (*p* < 0.050), ** (*p* < 0.010) and *** (*p* < 0.001) compared to 2D. (**b**) CD133, CD166, CD24, and CD90 protein expression of PC9 and PC9-GR3 models cultured on monolayer, 10% and 15%-PCL-ES scaffolds for 3 and 6 days. The 2D culture was used as an internal control and GAPDH as a loading control. The results shown are representative from at least three independent experiments.

The expression of CD133, CD166, CD24, and CD90 were evaluated in 3D culture by RT-qPCR and immunoblotting to determine the capacity of ES-PCL scaffolds to culture LCSC population (Figure 9). The uncropped Western blots can be found in Figure S4 and Figure S5.

Although *CD133* mRNA expression slightly increased in PC9 cultured on 3D matrices, a reduction was determined in its protein levels. The same results were found in CD24 mRNA and protein expression. *CD166* mRNA levels were greater in 3D in comparison with 2D, being statistically significant in 15%-PCL-ES supports after 3 days of culture and 10% and 15%-PCL ones after 6 days. CD166 protein levels were larger in PC9 cultured on 3D for 3 days, however they were reduced after 6 days of culture in contrast to the monolayer. CD90 mRNA and protein expression trended to decrease in cells seeded on PCL-ES scaffolds, except for 15%-PCL ones after 3 days of culture, which did not change their expression. 

*CD133* mRNA expression was slightly higher in PC9-GR3 grown on PCL-ES scaffolds compared to 2D after 3 days of culture. Nevertheless, a decrease in its protein levels was exhibited in cells cultured on 15%-PCL-ES meshes after 3 days and on both PCL-ES supports after 6 days. CD166 mRNA and protein expression were increased in 3D culture in comparison with the monolayer, being statistically significant in 10%-PCL-ES platforms. In contrast, CD24 mRNA and protein levels were larger in PC9-GR3 seeded on 10%-PCL-ES matrices, but did not change on 15%-PCL ones. No changes were observed in *CD90* in 3D after 3 days of culture, but a significant reduction was demonstrated after 6 days. These results are in agreement with CD90 protein levels.

#### 3.5.5. Hedgehog and Canonical Pathway of Sensitive and Resistant EGFRm Lung Adenocarcinoma Cell Models Cultured on PCL-ES Scaffolds

**Figure 10:**
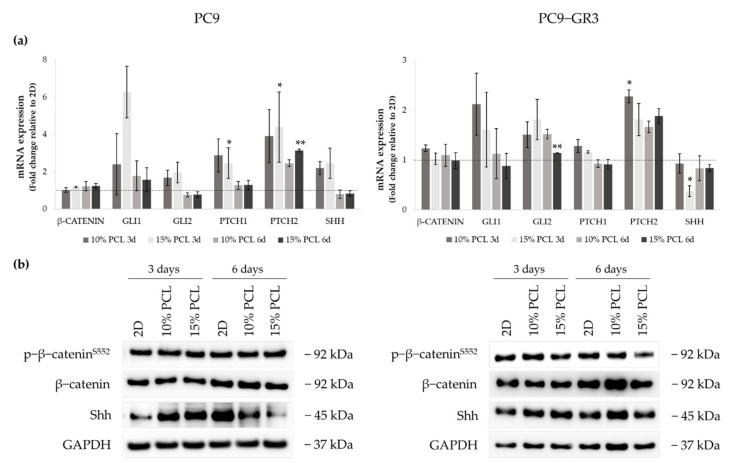
(**a**) *β-CATENIN*, *GLI1*, *GLI2*, *PTCH1*, *PTCH2*, and *SHH* mRNA levels of PC9 and PC9-GR3 cell models cultured on monolayer, 10% and 15%-PCL-ES scaffolds for 3 and 6 days. mRNA expression was normalized against the GAPDH gene. All cell culture conditions were compared to 2D, which was normalized to 1 (marked by the dotted line) and shown as fold change. The results are shown as mean ± SEM from at least three independent experiments. Levels of statistical significance are indicated as * (*p* < 0.050) and ** (*p* < 0.010) compared to 2D. (**b**) β-catenin and Shh protein expression of PC9 and PC9-GR3 models cultured on monolayer, 10% and 15%-PCL-ES scaffolds for 3 and 6 days. The 2D culture was used as an internal control and GAPDH as a loading control. The results shown are representative from at least three independent experiments.

We analyzed the role of the canonical (Wnt/β-catenin) and the Hedgehog signaling pathways in PC9 and PC9-GR3 cell models cultured on PCL-ES scaffolds for 3 and 6 days (Figure 10). The uncropped immunoblottings can be found in Figure S4 and Figure S5.

Regarding β-catenin mRNA and protein expression, no changes were exhibited in neither the cell model nor the cell culture condition. The mRNA expression of the transcriptional regulators *GLI1/2*, *PTCH1/2* receptors, and Sonic ligand *(SHH)* were increased in PC9 grown on PCL-ES meshes, being statistically significant in 15%-PCL ones for *PTCH1/2* compared to 2D after 3 days of culture. Nonetheless, no changes were found in *GLI1/2*, *PTCH1*, and *SHH* in 3D after 6 days of culture. *PTCH2* expression was larger in PC9 cultured on 3D for 6 days. Significant differences were observed in 15%-PCL ones in comparison with the monolayer. Shh protein levels were enhanced on cells seeded on PCL-ES supports, but then their levels were decreased after 6 days of culture.

Regarding changes in PC9-GR3, *GLI1* and *PTCH1* mRNA expression were greater in cells grown on 3D for 3 days in contrast to 2D. PCL-ES platforms caused an enhancement of *GLI2* and *PTCH2*. Significant differences were found in 15%-PCL-ES scaffolds after 6 days of culture for *GLI2* and in 10%-PCL ones after 3 days for *PTCH2* compared to the monolayer. No changes in *SHH* mRNA and its protein levels were exhibited, except for a significant reduction in 15%-PCL-ES structures after 3 days of culture.

### 3.6. CD133 and Vimentin Expression in EGFRm NSCLC Patient-Derived Tumors

#### 3.6.1. Patient and Tumor Characteristics

We analyzed data from 45 patients, who met inclusion criteria, with EGFRm NSCLC harboring exon 19 deletion and exon 21 L858R activating mutations (Table S3).

Regarding patients and tumor features, more than 75% were women with a median age of 68 years. Approximately 73% of patients were never smokers and most of them showed an ECOG of 0–1. The histology mainly identified was adenocarcinoma exhibiting a poor grade of differentiation. Almost 60% of the tumors harbored the exon 19 deletion and brain metastasis was observed in 20% of the patients before starting the treatment with the EGFR-TKI. Approximately 70% of the patients achieved a partial or a complete response to first or second generation EGFR-TKI.

#### 3.6.2. CD133 and Vimentin Tumor Expression in EGFRm NSCLC Patients

CD133 and Vimentin tumor expression were evaluated in 36 biopsies which had sufficient tumor sample for immunohistochemical analysis. The expression of CD133 and Vimentin were detected in 50% and 58% of tumor samples, respectively.

**Figure 11:**
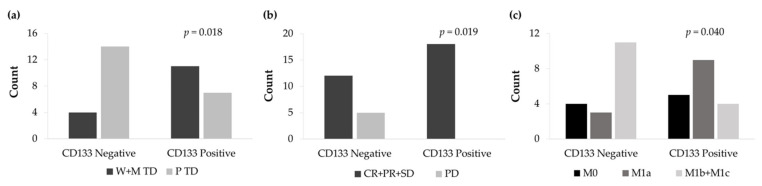
(**a**) Count of well and moderately tumor differentiation (W+M TD) and poorly tumor differentiation (P TD) cases according to the IHC expression levels of CD133. (**b**) Count of complete response, partial response, and stable disease (CR+PR+SD) and progression disease (PD) cases according to the IHC expression levels of CD133. (**c**) Count of non-metastasis (M0), local metastasis (M1a), and distant metastasis (M1b+M1c) cases according to the IHC expression levels of CD133.

The non-expression of the CD133 surface marker was significantly associated with a low degree of histological differentiation (*p* = 0.018) (Figure 11a). From all patients, 14 cases showed CD133 negative expression and poor tumor differentiation. In contrast, 11 CD133 positive patients exhibited well and moderate tumor differentiation. Furthermore, CD133 negative tumor expression significantly correlated with higher disease progression (*p* = 0.019) (Figure 11b) and a higher number of distant metastasis (*p* = 0.040) (Figure 11c).

**Figure 12:**
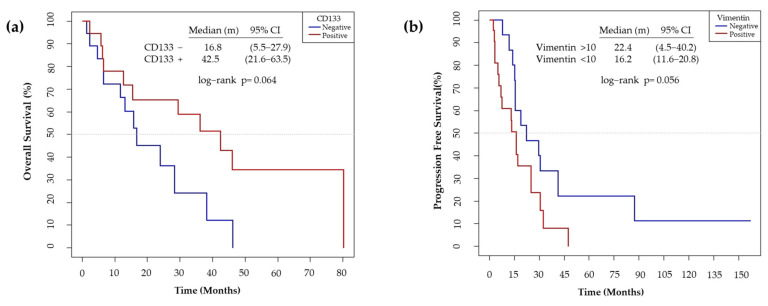
(**a**) Median overall survival according to the IHC expression levels of CD133. The non-expression of CD133 was considered as negative and the expression of > 1 %, as positive. (**b**) Median progression free survival according to the IHC expression levels of Vimentin. The expression of Vimentin <10% was considered as negative and >10%, as positive.

We also observed that the non-expression of CD133 showed a trend to worse OS (*p* = 0.064) (Figure 12a). On the other hand, high Vimentin tumor expression has a trend to a poor PFS (*p* = 0.056) (Figure 12b).

## 4. Discussion

Although researchers have developed several EGFR-TKIs for the treatment of EGFRm NSCLC, the majority of patients are diagnosed at advanced stages of the disease and eventually acquire resistance to the therapy through different mechanisms [2,6]. LCSCs have been identified as responsible for resistance to anticancer drugs, tumor relapse, and metastasis [7,8,9], but the research of this malignant subpopulation is not possible using monolayer cell culture [24,37]. Hence, different 3D culture strategies have been described to provide a more physiological environment for cells [26,28,32]. In this study, we evaluated the capacity of PCL-ES scaffolds to culture the LCSC niche in sensitive and resistant EGFRm lung adenocarcinoma cell models.

The use of PCL for biomedical applications has been increased over the years. Biocompatibility, bioresorbability, and low cost are very attractive features of this Food and Drug Administration (FDA) approved polymer [36]. Regarding the thermal characterization of PCL-ES matrices (Figure S1), the values obtained by TGA and DSC for weight degradation, T_m_, and ΔH_f_ of 3D structures coincide with the literature about PCL [41,42], and no differences were found between 10% and 15%-PCL-ES meshes. Stiffness and viscoelastic properties were also analyzed by DMA. An optimal stiffness of 3D structures is fundamental for cell adhesion, morphology, growth, and differentiation [43]. The E’ value for a healthy lung tissue is approximately 1.4 kPa, whereas that for polystyrene, the main component of 2D cell culture plates, is around 2100 MPa [44,45]. Thus, PCL-ES meshes are softer than 2D cell culture surfaces but stiffer than lung tissue, ranging from 4.52 to 5.40 MPa at 37 °C. According to the literature, the T_g_ for PCL is −60 °C, which is very similar to the values obtained for PCL-ES supports [41]. 

The spatial architecture is an important characteristic of 3D cell culture matrices. Through the analysis of SEM images, we calculated fiber diameter, porosity, and pore area of PCL-ES scaffolds (Table 2). The average filament diameter was 316 and 1764 nm for 10% and 15%-PCL-ES structures, respectively, which are in agreement with the literature [36,46,47,48,49,50]. A large porosity (approximately 90%) is recommended to provide a suitable space for cell attachment and a high-quality exchange of nutrients and metabolic waste [51,52]. On account of this, the porosity of 15%-PCL-ES meshes was 82%, which was significantly higher than 10%-PCL ones. Rabionet et al. demonstrated comparable findings with 7.5% and 15%-PCL-ES scaffolds [37]. Additionally, only 10%-PCL-ES supports showed beads. According to Nottelet and coworkers, beads were found in 7.5% and 9%-PCL-ES platforms, but not in 12% and 15%-PCL ones [49]. 

PCL-ES matrices increased their weight approximately 5% due to the sterilization process. In our study, we used overnight ethanol and 30 min of UV light. Guerra et al. concluded that the use of ethanol 70% for sterilization did not produce any significant effect on surface roughness, structure, distribution, and crystallinity of PCL structures, but it was observed a reduction of 11.9% in the Mw [53]. The soaking of PCL-ES meshes on RPMI-1640 for 28 days did not cause any change in their weight. Bölgen and colleagues described that PCL nanofibers with an average diameter of 196 nm reduced their elongation at break from 82 to 5.7% after 6 months in Ringer solution at 37 °C and pH 7.4 [54]. 

The protein adsorption on the surface of a scaffold strongly influences cell-scaffold interactions, determining cell attachment and proliferation [55]. Our findings showed that PCL-ES structures adsorb a great protein concentration (~0.8 and 1.2 g/L for 10% and 15%-PCL-ES meshes, respectively) after 3 days of incubation. Kumar et al. pointed out that the hydrophobic surfaces, i.e., PCL, were more covered by proteins than hydrophilic surfaces [56]. Besides this, no significant differences were found between both PCL-ES supports, which have similar surface roughness and chemistry, essential features for protein adsorption [57,58]. Nevertheless, a significant reduction in protein adsorption was also shown after 6 days of incubation due to the protein desorption rate and the exceeding of adsorption after a certain time [59].

Thereafter, PC9 and PC9-GR3 cell models were cultured on PCL-ES scaffolds for 3 and 6 days. Cell attachment to the 3D matrices was confirmed and the elongation of the nucleus in PC9-GR3 and the nucleus and cytoplasm in PC9 were determined in cells seeded on 15%-PCL-ES structures (Figure 2). Other researchers also found cell elongation on nanofibers in breast cancer cells [37] or fibroblasts [60]. In contrast, Moghadas et al. stated the formation of spheroids using highly hydrophobic ES meshes [32]. We also evaluated the expression of actin and tubulin (Figure 3). β-actin and γ-tubulin protein expression were reduced in PC9 cultured on 3D supports for 6 days, resulting in a more motile cell phenotype, oncogenic potential, and lower survival of NSCLC patients [61,62]. In PC9-GR3, α- and β-tubulin protein expression after 3 days and γ-tubulin after 6 days were upregulated on cells grown on PCL-ES scaffolds. High levels of βIII-tubulin have been associated with tumorigenic activity, chemoresistance, and poor survival of NSCLC patients [63,64]. 

The cell viability of PC9 and PC9-GR3 models cultured on PCL-ES structures were lower compared to monolayer (Figure 4). The same results were reported for different lung cancer cell lines seeded on decellularized lung scaffolds [26], chitosan–hyaluronic acid membranes [65], and gelatin meshes [66]. On the contrary, researchers proved that lung adenocarcinoma cell lines grown on silk/fibroin supports or AlgiMatrix^TM^ scaffolds showed higher viability compared to 2D [27,67]. Furthermore, cell models exhibited a significantly greater viability rate when cultured on 15%-PCL-ES platforms after 3 days. Pore size, surface availability, and porosity of 3D platforms as well as initial seeding cell density or time of culture, influence cell colonization [22,51]. The cell viability was also correlated to the protein adsorbed, which directly affected cell division [55,68].

Total EGFR protein levels were decreased in both cell models seeded on PCL-ES scaffolds (Figure 5), which are in agreement with the literature [69]. These results suggest a mechanism by which EGFRm lung adenocarcinoma cells acquire resistance to the EGFR-TKIs (Figure 6). Moreover, no changes were observed in phosphorylated EGFR expression in cells grown on PCL-ES platforms, probably as a consequence of the maintenance of LCSC features, for instance, self-renewal and pluripotency capacities [70,71]. The enrichment of the LCSC population was confirmed in sensitive and resistant lung adenocarcinoma models cultured on both PCL-ES scaffolds. According to Wang and coworkers, the identification of LCSCs can be carried out by three or more specific stem cell markers [72], which was fully accomplished in our study.

PC9 and PC9-GR3 cell models seeded on PCL-ES structures showed higher resistance to osimertinib (Figure 6), an irreversible small-molecule that binds covalently to the ATP-binding site of the tyrosine kinase domain of the EGFR and it is effective in the presence of activating mutations and T790M resistance mutation in the EGFR [6]. Additionally, a recent report proved that the LCSC niche was responsible for therapeutic resistance in NSCLC patients [73]. Previous studies demonstrated that lung cancer cells grown on AlgiMatrix^TM^ scaffolds [27], chitosan–hyaluronic acid membranes [65], or silk/fibroin structures [67] were less responsive to anticancer drugs in contrast to monolayer culture. PC9 cells cultured on PCL-ES supports also displayed upregulation of Nanog, which is also related to resistance to the treatment and tumor relapse and progression [74]. The multi-drug efflux pumps ABCB1 and ABCG2 are members of the ATP-binding cassette (ABC) family of transmembrane proteins [75]. Although ABCG2 has been commonly reported as a LCSC marker [71,76], our findings revealed enhanced levels of *ABCB1*. The expression of ABCB1 in patient samples has been linked to a poor response to chemotherapy [77,78]. In addition, several studies have associated the modulation of ABCB1 activity with the EGFR-TKIs [79,80]. Thus, the lower *ABCB1* enhancement found in PC9-GR3 compared to PC9 could be another intrinsic mechanism of resistance of PC9-GR3 [81]. 

Regarding the EMT process, both cell models cultured on PCL-ES meshes exhibited upregulation of Vimentin, *SNAIL*, and *TWIST* and downregulation of E-cadherin (Figure 7). The EMT process provides the capacity to metastasize by migrating from the primary tumor to another organ, and then, LCSCs can initiate another tumor [82,83]. Several researchers have observed that 3D culture using chitosan–hyaluronic acid matrices, spheroids, or hydrogel induced EMT through the modulation of different transcription factors and related proteins, such as Snail, Slug, Twist, Zeb1, E-cadherin, N-cadherin, or Fibronectin [84,85,86,87], which is consistent with our work. The EMT process has also been involved in the resistance to chemotherapy and EGFR-TKIs. PC9 cells seeded on decellularized lung scaffolds showed EMT induction and resistance to erlotinib and cisplatin [26]. PC9 grown on PCL-ES supports displayed a significantly greater *ZEB1* mRNA expression in comparison with the monolayer, which may lead to the resistance to osimertinib in the sensitive cell model (Figure 6) [88]. According to Tiran and colleagues, the activity of EMT transcription factors triggers LCSC genes, leading to cancer cell plasticity [89]. For instance, the induction of EMT results in decreased levels of CD24 [86,90], as shown in PC9 cultured on scaffolds (Figure 9). In addition, the softness of PCL-ES structures (Figure S1) influences EMT induction in sensitive and resistant lung adenocarcinoma models [91]. 

LCSCs possess self-renewal and pluripotency capacities that are usually controlled by Sox2, Oct-4, and Nanog [7,8,9,10,11,12]. However, Singh and Chellappan pointed out that Sox2 may regulate these capacities independently of Oct-4 and Nanog [8]. Sox2 is also related to high tumorigenic potential [92,93]. We observed increased levels of *SOX2*, Oct-4A, and p-Sox2 in PC9 and PC9-GR3 cell models grown on 3D meshes in comparison with 2D (Figure 8). An upregulation of Sox2 has also been found in lung cancer cells cultured on 3D cultures, such as chitosan–hyaluronan matrices [65,84] and spheroids [85]. The genomic amplification of Sox2 was detected in about 20% of lung adenocarcinoma patients and its high expression was significantly associated with a lower overall survival [93,94]. Furthermore, Sox2 overexpression has been linked to resistance to chemotherapy [73,85] and EGFR-TKIs [92]. Li and coworkers demonstrated that treatment with gefitinib causes the overexpression of Sox2 [95]. Furthermore, the continuous activation of EGFR leads to an increase in Sox2 expression, resulting in the maintenance of LCSC features in EGFRm lung adenocarcinoma [70,71]. 

Several surface markers have been described to identify LCSCs, such as CD133 and CD166 [14,15]. We observed upregulation of CD166 and reduced CD133 protein expression in both cell models cultured on PCL-ES supports (Figure 9). Different researchers found that CD166+ cells displayed self-renewal capacity and high tumorigenic activity [15,96,97]. Additionally, this cell population showed molecular signatures related to stem cells and biological functions related to angiogenesis, migration, or anti-apoptosis [15]. Zakaria et al. described that CD166+ cells overexpressed Sox2 and Oct-4A and interacted with the Hedgehog pathway [15]. Conversely, another study revealed that CD166 expression was associated with smaller tumors without lymph node metastasis [98]. Regarding CD133, the role of this marker remains unclear [72,99]. Different researchers demonstrated that CD133+ cells derived from NSCLC patients expressed high tumorigenic activity, enhanced levels of different stemness genes, high resistance to cisplatin, and self-renewal capacity [100,101]. Nevertheless, Zhang et al. pointed out that no differences in the tumorigenic activity were found between CD133- and CD133+ populations from NSCLC patient samples using a more sensitive mouse xenotransplantation assay [97]. Other studies discovered that CD133- and CD133+ populations had the same self-renewal and tumorigenic capacities [102,103]. Additionally, the CD133+ population was sensitive to the EGFR-TKI afatinib in EGFRm lung adenocarcinoma H1650 and H1975 cell lines [104]. The variability of the CD133 marker in the aforementioned studies may be a consequence of the heterogeneity of NSCLC. Although the samples were classified by histologic subtypes, mutations in oncogenes were not taken into account, which may influence the results.

The Hedgehog signaling pathway plays an important role in the development and repair of normal lung tissue [105]. Researchers have related its inhibition to the loss of LCSC properties [21]. We also found the activation of the Hedgehog pathway in PC9 and PC9-GR3 cell models cultured on PCL-ES scaffolds (Figure 10). A recent study reported that 76% of lung adenocarcinoma patients express GLI1, the amplifier of the pathway [106]. Besides this, GLI1 also supports the EMT process leading to resistance to EGFR-TKIs in EGFRm lung adenocarcinoma [107,108,109]. Schnidar et al. demonstrated that an oncogenic transformation could be induced by the synergic activation of EGFR/MAPK and Hedgehog/GLI1 pathways [110]. The Hedgehog pathway and EGFR co-stimulate LCSC markers, e.g., Sox2 [109]. While PC9 seeded on 3D structures exhibited an upregulation of Shh after 3 days of culture, no changes were observed in PC9-GR3. According to Lauth et al., other pathways such as RAS, TGFβ, and PI3K can activate the non-canonical Hedgehog pathway, inducing GLI expression [111]. 

Taking all this into account, 15%-PCL-ES scaffolds may be a better 3D strategy than 10%-PCL ones. Cells cultured on 15%-PCL-ES structures exhibited a higher cell elongation and cell viability (Figure 2; Figure 4). Some LCSC properties were also significantly upregulated only in 15%-PCL-ES supports compared to monolayer, such as *SNAIL* in PC9 and PC9-GR3 models, *TWIST* in PC9, and *SLUG* in PC9-GR3 (Figure 7a). Another example is the CD133 reduction in PC9 and PC9-GR3, which was more notable in cells grown on 15%-PCL-ES platforms for 3 days (Figure 9b). Although the behavior of PC9 and PC9-GR3 cultured on PCL-ES matrices were similar, some variations were found in the EMT process and stemness and pluripotency capacities (Figure 7; Figure 8). Differences between cells seeded on 2D and PCL-ES meshes were already observed after 3 days of culture in PC9. However, PC9-GR3 grown on 3D matrices required 6 days to express these changes. Resistance to EGFR-TKIs has been linked to EMT and stemness and pluripotency capacities [88,92]. PC9-GR3 may already have a baseline upregulation of these properties and more days were needed to show differences between monolayer and 3D culture.

Patients’ data analysis agrees with the literature about NSCLC patients harboring EGFR sensitive mutations in the European population [112]. Hence, our sample is representative of the patient profile and tumor type. High levels of Vimentin were associated with lower PFS in our cohort of patients (Figure 12b). Previous studies observed that high expression of Vimentin was associated with a poor outcome to first generation EGFR-TKI and the development of brain metastasis in EGFRm NSCLC patients [113,114]. Our findings also revealed that non-expression of CD133 was associated with a poor degree of histological differentiation, progression to the disease, distant metastasis (Figure 11), and a low OS (Figure 12a). Wen and coworkers determined that high *CD133* mRNA levels were related to a better survival rate in lung cancer [115]. Different studies concluded that there was no relationship between CD133 expression and prognosis in NSCLC patients [116,117]. Conversely, other studies established CD133 as an independent prognostic marker for NSCLC [118,119]. Nevertheless, to the best of our knowledge, basal CD133 expression levels in NSCLC patients harboring EGFR sensitivity mutations have not been previously investigated. CD133+ expression was associated with a better prognosis in EGFRm NSCLC patients. Our study has demonstrated that the use of PCL-ES scaffolds allows the enrichment of LCSCs, which are associated with cancer recurrence, resistance to therapies, and metastasis [7,8,9]. Cells cultured on these 3D supports exhibited higher levels of Vimentin (Figure 7) and lower expression of CD133 (Figure 9) compared to 2D. Taking into account in vitro results, the behavior of cells seeded on PCL-ES scaffolds is more similar to the results found in patients (Figure 11; Figure 12). The following limitations in our study may have influenced the results. First, it was a retrospective study with the biases that this entails. Second, the number of samples with enough tissue available to perform IHC was less than expected, and third, some tumor samples were quite old, which could modify the IHC results. However, in relation to this issue, the percentage of Vimentin expression observed in our samples is consistent with that reported in previous studies [113].

## 5. Conclusions

PCL-ES scaffolds are useful for the 3D cell culture of EGFRm lung adenocarcinoma cell models. The 3D structures displayed different properties that support cell attachment, proliferation, and morphology changes. Consequently, cell models grown on PCL-ES matrices amplified several LCSC characteristics. We showed higher resistance to osimertinib, upregulation of drug efflux pumps, EMT process, stemness, and surface markers, and the activation of the Hedgehog pathway. Additionally, our study demonstrated that the lack of CD133 expression is related to the LCSC population. In vitro, we observed a downregulation of CD133 protein expression when the LCSC niche was enriched. Moreover, in tumor tissue samples of EGFRm NSCLC patients, the non-expression of CD133 was significantly associated with a low degree of histological differentiation, progression of the disease, and distant metastasis, features directly connected to LCSCs. Regarding the results of Vimentin, the same correlation was revealed between in vitro and IHC patient results. Therefore, we conclude that the use of PCL-ES scaffolds for culturing EGFRm lung adenocarcinoma cell models is a trustworthy 3D strategy to simulate physiological conditions allowing the study of this lung cancer subtype in order to find new biomarkers or test new drugs.

## Figures and Tables

**Table 1:** Pictures from the top and bottom sides of 10% and 15%-PCL-ES scaffolds at different magnifications displayed by scanning electronic microscopy (SEM).

	Side	Magnification	
		×1000	×5000
**10% PCL**	Top	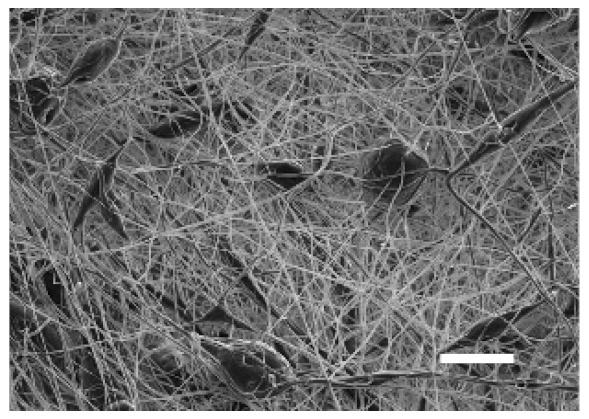	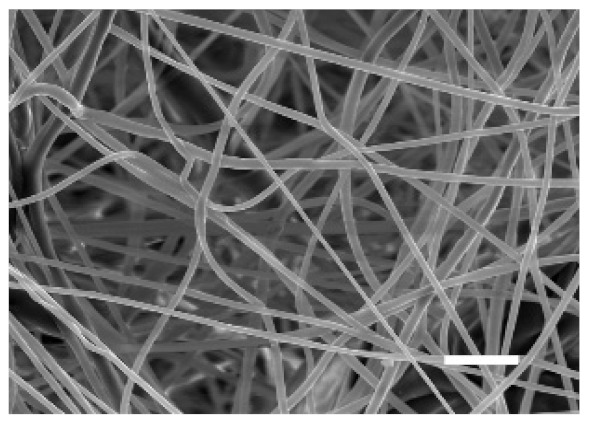
	Bottom	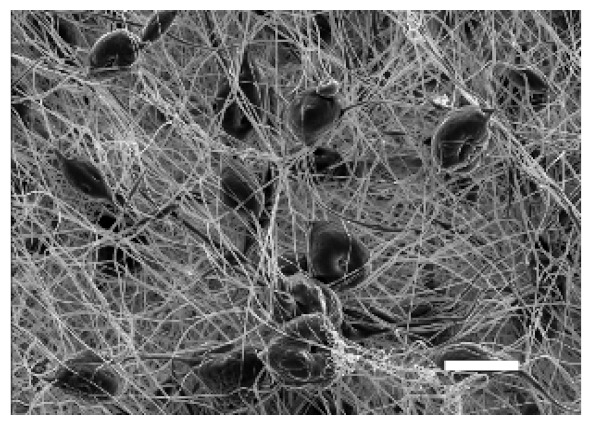	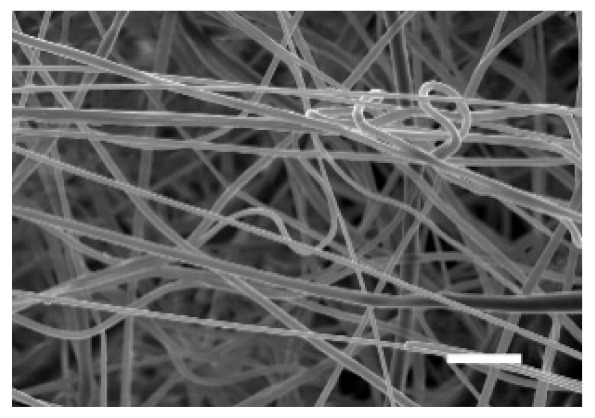
**15% PCL**	Top	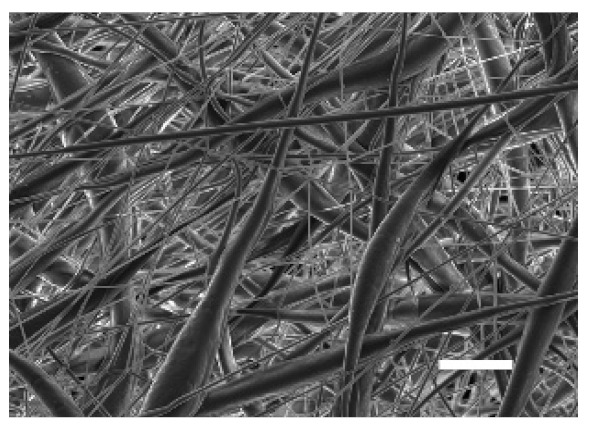	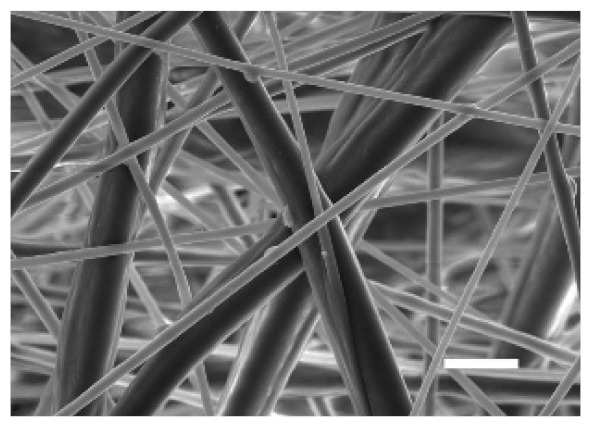
	Bottom	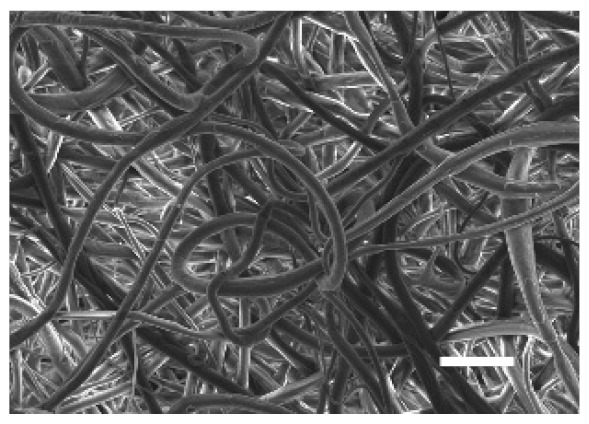	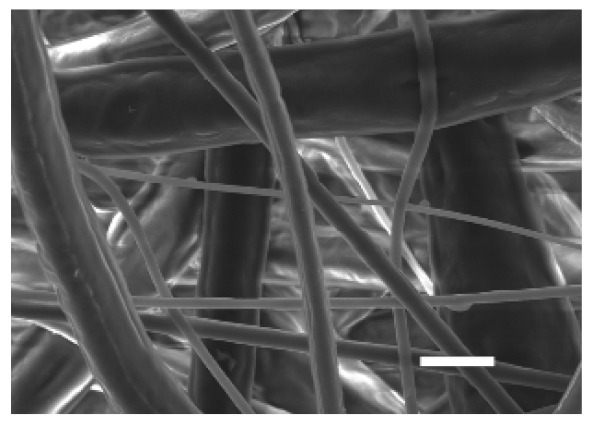
		Scale bars: 15 µm	Scale bars: 3 µm

**Table 2:** Filament diameter, surface porosity, and pore area of 10% and 15%-PCL-ES scaffolds. Images from the top and bottom sides were used to calculate the parameters. The results are shown as mean ± SEM. Levels of statistical significance are indicated as *** (*p* > 0.001).

Parameter	10% PCL	15% PCL
Filament diameter (nm)	315.67 ± 21.84	1764.42 ± 333.43 ^(***)^
Surface Porosity (%)	68.26 ± 1.06	82.29 ± 3.68 ^(***)^
Pore Area (µm^2^)	0.36 ± 0.13	0.73 ± 0.25 ^(***)^

## Data Availability

The data presented in this study are available in this article (and Supplementary Materials).

## Supplementary Materials

The following are available online at https://www.mdpi.com/article/10.3390/cancers13215320/s1. Figure S1: Thermogravimetric analysis of (a) 10%-PCL-ES scaffolds and (b) 15%-PCL-ES scaffolds. Differential scanning calorimetry of (c) 10%-PCL-ES scaffolds and (d) 15%-PCL-ES scaffolds. Dynamic mechanical analysis of (e) 10%-PCL-ES scaffolds and (f) 15%-PCL-ES scaffolds; Figure S2: Filament diameter histogram of (a) 10%-PCL-ES scaffolds and (b) 15%-PCL-ES scaffolds; Figure S3: Whole Western blot figures from Figure 3b and 5 showing α-tubulin, β-tubulin, γ-tubulin, β-actin, p-EGFR, EGFR, and GAPDH protein bands with molecular weight markers (merge of colorimetric and chemiluminescence) of PC9 and PC9-GR3; Figure S4: Whole Western blot figures protein bands of (a) Figure 7b, (b) Figure 8b, (c) Figure 9b, and (d) Figure 10b with molecular weight markers (merge of colorimetric and chemiluminescence) of PC9; Figure S5: Whole Western blot figures protein bands of (a) Figure 7b, (b) Figure 8b, (c) Figure 9b, and (d) Figure 10b with molecular weight markers (merge of colorimetric and chemiluminescence) of PC9-GR3; Table S1: Primer design; Table S2: List of antibodies; Table S3: Patient and tumor characteristics at baseline.

## References

[1] Bray F., Ferlay J., Soerjomataram I., Siegel R.L., Torre L.A., Jemal A. (2018). Global Cancer Statistics 2018: GLOBOCAN Estimates of Incidence and Mortality Worldwide for 36 Cancers in 185 Countries. CA Cancer J. Clin..

[2] Bade B.C., Dela Cruz C.S. (2020). Lung Cancer 2020: Epidemiology, Etiology, and Prevention. Clin. Chest Med..

[3] Travis W.D., Brambilla E., Nicholson A.G., Yatabe Y., Austin J.H.M., Beth Beasley M., Chirieac L.R., Dacic S., Duhig E., Flieder D.B. (2015). The 2015 World Health Organization Classification of Lung Tumors. J. Thorac. Oncol..

[4] Forsythe M.L., Alwithenani A., Bethune D., Castonguay M., Drucker A., Flowerdew G., French D., Fris J., Greer W., Henteleff H. (2020). Molecular profiling of non-small cell lung cancer. PLoS ONE.

[5] Lynch T.J., Bell D.W., Sordella R., Gurubhagavatula S., Okimoto R.A., Brannigan B.W., Harris P.L., Haserlat S.M., Supko J.G., Haluska F.G. (2004). Activating Mutations in the Epidermal Growth Factor Receptor Underlying Responsiveness of Non-Small-Cell Lung Cancer to Gefitinib. N. Engl. J. Med..

[6] Wu S.G., Shih J.Y. (2018). Management of acquired resistance to EGFR TKI-targeted therapy in advanced non-small cell lung cancer. Mol. Cancer.

[7] Yu Y., Ramena G., Elble R.C. (2012). The role of cancer stem cells in relapse of solid tumors. Front. Biosci..

[8] Singh S., Chellappan S. (2014). Lung cancer stem cells: Molecular features and therapeutic targets. Mol. Aspects Med..

[9] Medema J.P. (2013). Cancer stem cells: The challenges ahead. Nat. Cell Biol..

[10] Hyslop L., Stojkovic M., Armstrong L., Walter T., Stojkovic P., Przyborski S., Herbert M., Murdoch A., Strachan T., Lako M. (2005). Downregulation of NANOG Induces Differentiation of Human Embryonic Stem Cells to Extraembryonic Lineages. Stem Cells.

[11] Sarkar A., Hochedlinger K. (2013). The Sox family of transcription factors: Versatile regulators of stem and progenitor cell fate. Cell Stem Cell.

[12] Kumar S.M., Liu S., Lu H., Zhang H., Zhang P.J., Gimotty P.A., Guerra M., Guo W., Xu X. (2012). Acquired cancer stem cell phenotypes through Oct4-mediated dedifferentiation. Oncogene.

[13] Nassar D., Blanpain C. (2016). Cancer Stem Cells: Basic Concepts and Therapeutic Implications. Annu. Rev. Pathol. Mech. Dis..

[14] Zhang D.G., Jiang A.G., Lu H.Y., Zhang L.X., Gao X.Y. (2015). Isolation, cultivation and identification of human lung adenocarcinoma stem cells. Oncol. Lett..

[15] Zakaria N., Yusoff N.M., Zakaria Z., Lim M.N., Baharuddin P.J.N., Fakiruddin K.S., Yahaya B. (2015). Human non-small cell lung cancer expresses putative cancer stem cell markers and exhibits the transcriptomic profile of multipotent cells. BMC Cancer.

[16] Akunuru S., Zhai Q.J., Zheng Y. (2012). Non-small cell lung cancer stem/progenitor cells are enriched in multiple distinct phenotypic subpopulations and exhibit plasticity. Cell Death Dis..

[17] Yan X., Luo H., Zhou X., Zhu B., Wang Y., Bian X. (2013). Identification of CD90 as a marker for lung cancer stem cells in A549 and H446 cell lines. Oncol. Rep..

[18] Zhang Y., Weinberg R.A. (2018). Epithelial-to-mesenchymal transition in cancer: Complexity and opportunities. Front. Med..

[19] Pastushenko I., Brisebarre A., Sifrim A., Fioramonti M., Revenco T., Boumahdi S., Van Keymeulen A., Brown D., Moers V., Lemaire S. (2018). Identification of the tumour transition states occurring during EMT. Nature.

[20] Teng Y., Wang X., Wang Y., Ma D. (2010). Wnt/β-catenin signaling regulates cancer stem cells in lung cancer A549 cells. Biochem. Biophys. Res. Commun..

[21] Park K.S., Martelotto L.G., Peifer M., Sos M.L., Karnezis A.N., Mahjoub M.R., Bernard K., Conklin J.F., Szczepny A., Yuan J. (2011). A crucial requirement for Hedgehog signaling in small cell lung cancer. Nat. Med..

[22] Costard L.S., Hosn R.R., Ramanayake H., O’Brien F.J., Curtin C.M. (2021). Influences of the 3D microenvironment on cancer cell behaviour and treatment responsiveness: A recent update on lung, breast and prostate cancer models. Acta Biomater..

[23] Boucherit N., Gorvel L., Olive D. (2020). 3D Tumor Models and Their Use for the Testing of Immunotherapies. Front. Immunol..

[24] Chitcholtan K., Asselin E., Parent S., Sykes P.H., Evans J.J. (2013). Differences in growth properties of endometrial cancer in three dimensional (3D) culture and 2D cell monolayer. Exp. Cell Res..

[25] Mak I.W., Evaniew N., Ghert M. (2014). Lost in translation: Animal models and clinical trials in cancer treatment. Am. J. Transl. Res..

[26] Tapias L.F., Gilpin S.E., Ren X., Wei L., Fuchs B.C., Tanabe K.K., Lanuti M., Ott H.C. (2015). Assessment of Proliferation and Cytotoxicity in a Biomimetic Three-Dimensional Model of Lung Cancer. Ann. Thorac. Surg..

[27] Godugu C., Patel A.R., Desai U., Andey T., Sams A., Singh M. (2013). AlgiMatrix^TM^ Based 3D Cell Culture System as an In-Vitro Tumor Model for Anticancer Studies. PLoS ONE.

[28] Yao K., Li W., Li K., Wu Q., Gu Y., Zhao L., Zhang Y., Gao X. (2020). Simple Fabrication of Multicomponent Heterogeneous Fibers for Cell Co-Culture via Microfluidic Spinning. Macromol. Biosci..

[29] Sachs N., Papaspyropoulos A., Zomer-van Ommen D.D., Heo I., Böttinger L., Klay D., Weeber F., Huelsz-Prince G., Iakobachvili N., Amatngalim G.D. (2019). Long-term expanding human airway organoids for disease modeling. EMBO J..

[30] Nakamura H., Sugano M., Miyashita T., Hashimoto H., Ochiai A., Suzuki K., Tsuboi M., Ishii G. (2019). Organoid culture containing cancer cells and stromal cells reveals that podoplanin-positive cancer-associated fibroblasts enhance proliferation of lung cancer cells. Lung Cancer.

[31] Herreros-Pomares A., Zhou X., Calabuig-Fariñas S., Lee S.-J., Torres S., Esworthy T., Hann S.Y., Jantus-Lewintre E., Camps C., Zhang L.G. (2021). 3D printing novel in vitro cancer cell culture model systems for lung cancer stem cell study. Mater. Sci. Eng. C.

[32] Moghadas H., Saidi M.S., Kashaninejad N., Kiyoumarsioskouei A., Nguyen N.T. (2017). Fabrication and characterization of low-cost, bead-free, durable and hydrophobic electrospun membrane for 3D cell culture. Biomed. Microdevices.

[33] Chen S., Giannakou A., Wyman S., Gruzas J., Golas J., Zhong W., Loreth C., Sridharan L., Yamin T.-T., Damelin M. (2018). Cancer-associated fibroblasts suppress SOX2-induced dysplasia in a lung squamous cancer coculture. Proc. Natl. Acad. Sci. USA.

[34] Hu S., Dasbiswas K., Guo Z., Tee Y.H., Thiagarajan V., Hersen P., Chew T.L., Safran S.A., Zaidel-Bar R., Bershadsky A.D. (2017). Long-range self-organization of cytoskeletal myosin II filament stacks. Nat. Cell Biol..

[35] Reneker D.H., Chun I. (1996). Nanometre diameter fibres of polymer, produced by electrospinning. Nanotechnology.

[36] Cipitria A., Skelton A., Dargaville T.R., Dalton P.D., Hutmacher D.W. (2011). Design, fabrication and characterization of PCL electrospun scaffolds—A review. J. Mater. Chem..

[37] Rabionet M., Yeste M., Puig T., Ciurana J. (2017). Electrospinning PCL Scaffolds Manufacture for Three-Dimensional Breast Cancer Cell Culture. Polymers.

[38] Polonio-Alcalá E., Rabionet M., Guerra A., Yeste M., Ciurana J., Puig T., Polonio-Alcalá E., Rabionet M., Guerra A.J., Yeste M. (2018). Screening of Additive Manufactured Scaffolds Designs for Triple Negative Breast Cancer 3D Cell Culture and Stem-Like Expansion. Int. J. Mol. Sci..

[39] Polonio-Alcalá E., Rabionet M., Gallardo X., Angelats D., Ciurana J., Ruiz-Martínez S., Puig T. (2019). PLA Electrospun Scaffolds for Three-Dimensional Triple-Negative Breast Cancer Cell Culture. Polymers.

[40] Saha S., Duan X., Wu L., Lo P.-K., Chen H., Wang Q. (2012). Electrospun fibrous scaffolds promote breast cancer cell alignment and epithelial-mesenchymal transition. Langmuir.

[41] Mohamed A., Gordon S.H., Biresaw G. (2007). Polyaprolactone/polystyrene bioblends characterized by thermogravimetry, modulated differential scanning calorimetry and infrared photoacoustic spectroscopy. Polym. Degrad. Stab..

[42] Wang Y., Rodriguez-Perez M.A., Reis R.L., Mano J.F. (2005). Thermal and thermomechanical behaviour of polycaprolactone and starch/polycaprolactone blends for biomedical applications. Macromol. Mater. Eng..

[43] Balgude A.P., Yu X., Szymanski A., Bellamkonda R.V. (2001). Agarose gel stiffness determines rate of DRG neurite extension in 3D cultures. Biomaterials.

[44] Singh D., Zo S.M., Kumar A., Han S.S. (2013). Engineering three-dimensional macroporous hydroxyethyl methacrylate- alginate-gelatin cryogel for growth and proliferation of lung epithelial cells. J. Biomater. Sci. Polym. Ed..

[45] Russo P., Acierno D., Corradi A., Leonelli C. (2011). Dynamic-mechanical behavior and morphology of polystyrene/perovskite composites: Effects of filler size. Procedia Eng..

[46] Wu H., Fan J., Chu C.C., Wu J. (2010). Electrospinning of small diameter 3-D nanofibrous tubular scaffolds with controllable nanofiber orientations for vascular grafts. J. Mater. Sci. Mater. Med..

[47] Shin M., Ishii O., Sueda T., Vacanti J.P. (2004). Contractile cardiac grafts using a novel nanofibrous mesh. Biomaterials.

[48] Ishii O., Shin M., Sueda T., Vacanti J.P. (2005). In vitro tissue engineering of a cardiac graft using a degradable scaffold with an extracellular matrix-like topography. J. Thorac. Cardiovasc. Surg..

[49] Nottelet B., Pektok E., Mandracchia D., Tille J.C., Walpoth B., Gurny R., Möller M. (2009). Factorial design optimization and in vivo feasibility of poly(ε-caprolactone)-micro- and nanofiber-based small diameter vascular grafts. J. Biomed. Mater. Res. Part A.

[50] Pektok E., Nottelet B., Tille J.C., Gurny R., Kalangos A., Moeller M., Walpoth B.H. (2008). Degradation and healing characteristics of small-diameter poly(ε-caprolactone) vascular grafts in the rat systemic arterial circulation. Circulation.

[51] Lawrence B.J., Madihally S.V. (2008). Cell colonization in degradable 3D porous matrices. Cell Adhes. Migr..

[52] Li W.J., Laurencin C.T., Caterson E.J., Tuan R.S., Ko F.K. (2002). Electrospun nanofibrous structure: A novel scaffold for tissue engineering. J. Biomed. Mater. Res..

[53] Guerra A.J., Cano P., Rabionet M., Puig T., Ciurana J. (2018). Effects of different sterilization processes on the properties of a novel 3D-printed polycaprolactone stent. Polym. Adv. Technol..

[54] Bölgen N., Menceloǧlu Y.Z., Acatay K., Vargel I., Pişkin E. (2005). In vitro and in vivo degradation of non-woven materials made of poly(ε-caprolactone) nanofibers prepared by electrospinning under different conditions. J. Biomater. Sci. Polym. Ed..

[55] Firkowska-Boden I., Zhang X., Jandt K.D. (2018). Controlling Protein Adsorption through Nanostructured Polymeric Surfaces. Adv. Healthc. Mater..

[56] Kumar N., Parajuli O., Gupta A., Hahm J.I. (2008). Elucidation of protein adsorption behavior on polymeric surfaces: Toward high-density, high-payload protein templates. Langmuir.

[57] Nandakumar A., Tahmasebi Birgani Z., Santos D., Mentink A., Auffermann N., Van Der Werf K., Bennink M., Moroni L., Van Blitterswijk C., Habibovic P. (2013). Surface modification of electrospun fibre meshes by oxygen plasma for bone regeneration. Biofabrication.

[58] Santos M.I., Pashkuleva I., Alves C.M., Gomes M.E., Fuchs S., Unger R.E., Reis R.L., Kirkpatrick C.J. (2009). Surface-modified 3D starch-based scaffold for improved endothelialization for bone tissue engineering. J. Mater. Chem..

[59] Smith J. (1981). Chemical Engineering Kinetics.

[60] Li D., Wu T., He N., Wang J., Chen W., He L., Huang C., EI-Hamshary H.A., Al-Deyab S.S., Ke Q. (2014). Three-dimensional polycaprolactone scaffold via needleless electrospinning promotes cell proliferation and infiltration. Colloids Surfaces B Biointerfaces.

[61] Dugina V., Khromova N., Rybko V., Blizniukov O., Shagieva G., Chaponnier C., Kopnin B., Kopnin P. (2015). Tumor promotion by γ and suppression by β non-muscle actin isoforms. Oncotarget.

[62] Maounis N.F., Dráberová E., Trakas N., Chorti M., Riga D., Tzannis K., Kanakis M., Voralu K., Ellina E., Mahera E. (2018). Expression of γ-tubulin in non-small cell lung cancer and effect on patient survival. Histol. Histopathol..

[63] Levallet G., Bergot E., Antoine M., Creveuil C., Santos A.O., Beau-Faller M., De Fraipont F., Brambilla E., Levallet J., Morin F. (2012). High TUBB3 expression, an independent prognostic marker in patients with early non-small cell lung cancer treated by preoperative chemotherapy, is regulated by K-ras signaling pathway. Mol. Cancer Ther..

[64] McCarroll J.A., Gan P.P., Liu M., Kavallaris M. (2010). βIII-tubulin is a multifunctional protein involved in drug sensitivity and tumorigenesis in non-small cell lung cancer. Cancer Res..

[65] Huang Y.J., Hsu S. (2014). Acquisition of epithelial-mesenchymal transition and cancer stem-like phenotypes within chitosan-hyaluronan membrane-derived 3D tumor spheroids. Biomaterials.

[66] Yan X., Zhou L., Wu Z., Wang X., Chen X., Yang F., Guo Y., Wu M., Chen Y., Li W. (2019). High throughput scaffold-based 3D micro-tumor array for efficient drug screening and chemosensitivity testing. Biomaterials.

[67] Li J., Zhou Y., Chen W., Yuan Z., You B., Liu Y., Yang S., Li F., Qu C., Zhang X. (2018). A Novel 3D in Vitro Tumor Model Based on Silk Fibroin/Chitosan Scaffolds to Mimic the Tumor Microenvironment. ACS Appl. Mater. Interfaces.

[68] Baxter G.C., Stanners C.P. (1978). The effect of protein degradation on cellular growth characteristics. J. Cell. Physiol..

[69] Ekert J.E., Johnson K., Strake B., Pardinas J., Jarantow S., Perkinson R., Colter D.C. (2014). Three-dimensional lung tumor microenvironment modulates therapeutic compound responsiveness in vitro--implication for drug development. PLoS ONE.

[70] Du W., Ni L., Liu B., Wei Y., Lv Y., Qiang S., Dong J., Liu X. (2018). Upregulation of SALL4 by EGFR activation regulates the stemness of CD44-positive lung cancer. Oncogenesis.

[71] Singh S., Trevino J., Bora-Singhal N., Coppola D., Haura E., Altiok S., Chellappan S.P. (2012). EGFR/Src/Akt signaling modulates Sox2 expression and self-renewal of stem-like side-population cells in non-small cell lung cancer. Mol. Cancer.

[72] Wang Y., Jiang M., Du C., Yu Y., Liu Y., Li M., Luo F. (2018). Utilization of lung cancer cell lines for the study of lung cancer stem cells. Oncol. Lett..

[73] Wang L., Liu X., Ren Y., Zhang J., Chen J., Zhou W., Guo W., Wang X., Chen H., Li M. (2017). Cisplatin-enriching cancer stem cells confer multidrug resistance in non-small cell lung cancer via enhancing TRIB1/HDAC activity. Cell Death Dis..

[74] Gong S., Li Q., Jeter C.R., Fan Q., Tang D.G., Liu B. (2015). Regulation of NANOG in cancer cells. Mol. Carcinog..

[75] Robey R.W., Pluchino K.M., Hall M.D., Fojo A.T., Bates S.E., Gottesman M.M. (2018). Revisiting the role of ABC transporters in multidrug-resistant cancer. Nat. Rev. Cancer.

[76] Liu Y.P., Yang C.J., Huang M.S., Yeh C.T., Wu A.T.H., Lee Y.C., Lai T.C., Lee C.H., Hsiao Y.W., Lu J. (2013). Cisplatin selects for multidrug-resistant CD133+ cells in lung adenocarcinoma by activating notch signaling. Cancer Res..

[77] Amiri-Kordestani L., Basseville A., Kurdziel K., Fojo A.T., Bates S.E. (2012). Targeting MDR in breast and lung cancer: Discriminating its potential importance from the failure of drug resistance reversal studies. Drug Resist. Updat..

[78] Goldstein L.J., Galski H., Fojo A., Willingham M., Lai S.L., Gazdar A., Pirker R., Green A., Crist W., Brodeur G.M. (1989). Expression of multidrug resistance gene in human cancers. J. Natl. Cancer Inst..

[79] Liu B., Guo Z., Dong H., Daofeng T., Cai Q., Ji B., Zhang S., Wu L., Wang J., Wang L. (2015). LRIG1, human EGFR inhibitor, reverses multidrug resistance through modulation of ABCB1 and ABCG2. Brain Res..

[80] Shi Z., Tiwari A.K., Shukla S., Robey R.W., Kim I.W., Parmar S., Bates S.E., Si Q.S., Goldblatt C.S., Abraham I. (2009). Inhibiting the function of ABCB1 and ABCG2 by the EGFR tyrosine kinase inhibitor AG1478. Biochem. Pharmacol..

[81] Jacobsen K., Bertran-Alamillo J., Molina M.A., Teixidó C., Karachaliou N., Pedersen M.H., Castellví J., Garzón M., Codony-Servat C., Codony-Servat J. (2017). Convergent Akt activation drives acquired EGFR inhibitor resistance in lung cancer. Nat. Commun..

[82] Nieto M.A. (2009). Epithelial-Mesenchymal Transitions in development and disease: Old views and new perspectives. Int. J. Dev. Biol..

[83] Li S., Li Q. (2014). Cancer stem cells and tumor metastasis (review). Int. J. Oncol..

[84] Han H.-W., Hsu S. (2016). Chitosan-hyaluronan based 3D co-culture platform for studying the crosstalk of lung cancer cells and mesenchymal stem cells. Acta Biomater..

[85] Choe C., Kim H., Min S., Park S., Seo J., Roh S. (2018). SOX2, a stemness gene, induces progression of NSCLC A549 cells toward anchorage-independent growth and chemoresistance to vinblastine. Onco. Targets. Ther..

[86] Liu P., Zhang R., Yu W., Ye Y., Cheng Y., Han L., Dong L., Chen Y., Wei X., Yu J. (2017). FGF1 and IGF1-conditioned 3D culture system promoted the amplification and cancer stemness of lung cancer cells. Biomaterials.

[87] Kumar M., Allison D.F., Baranova N.N., Wamsley J.J., Katz A.J., Bekiranov S., Jones D.R., Mayo M.W. (2013). NF-κB Regulates Mesenchymal Transition for the Induction of Non-Small Cell Lung Cancer Initiating Cells. PLoS ONE.

[88] Song K.A., Niederst M.J., Lochmann T.L., Hata A.N., Kitai H., Ham J., Floros K.V., Hicks M.A., Hu H., Mulvey H.E. (2018). Epithelial-to-mesenchymal transition antagonizes response to targeted therapies in lung cancer by suppressing BIM. Clin. Cancer Res..

[89] Tiran V., Lindenmann J., Brcic L., Heitzer E., Stanzer S., Tabrizi-Wizsy N.G., Stacher E., Stoeger H., Popper H.H., Balic M. (2017). Primary patient-derived lung adenocarcinoma cell culture challenges the association of cancer stem cells with epithelial-to-mesenchymal transition. Sci. Rep..

[90] Mani S.A., Guo W., Liao M.J., Eaton E.N., Ayyanan A., Zhou A.Y., Brooks M., Reinhard F., Zhang C.C., Shipitsin M. (2008). The Epithelial-Mesenchymal Transition Generates Cells with Properties of Stem Cells. Cell.

[91] Mihalko E.P., Brown A.C. (2018). Material Strategies for Modulating Epithelial to Mesenchymal Transitions. ACS Biomater. Sci. Eng..

[92] Murakami A., Takahashi F., Nurwidya F., Kobayashi I., Minakata K., Hashimoto M., Nara T., Kato M., Tajima K., Shimada N. (2014). Hypoxia increases gefitinib-resistant lung cancer stem cells through the activation of insulin-like growth factor 1 receptor. PLoS ONE.

[93] Nakatsugawa M., Takahashi A., Hirohashi Y., Torigoe T., Inoda S., Murase M., Asanuma H., Tamura Y., Morita R., Michifuri Y. (2011). SOX2 is overexpressed in stem-like cells of human lung adenocarcinoma and augments the tumorigenicity. Lab. Investig..

[94] Sholl L.M., Barletta J.A., Yeap B.Y., Chirieac L.R., Hornick J.L. (2010). Sox2 protein expression is an independent poor prognostic indicator in stage i lung adenocarcinoma. Am. J. Surg. Pathol..

[95] Li M., Yang J., Zhou W., Ren Y., Wang X., Chen H., Zhang J., Chen J., Sun Y., Cui L. (2017). Activation of an AKT/FOXM1/STMN1 pathway drives resistance to tyrosine kinase inhibitors in lung cancer. Br. J. Cancer.

[96] Jiang Z., Hao Y., Ding X., Zhang Z., Liu P., Wei X., Xi J. (2016). The effects and mechanisms of SLC34A2 on tumorigenicity in human non-small cell lung cancer stem cells. Tumor Biol..

[97] Zhang W.C., Ng S.C., Yang H., Rai A., Umashankar S., Ma S., Soh B.S., Sun L.L., Tai B.C., Nga M.E. (2012). Glycine decarboxylase activity drives non-small cell lung cancer tumor-initiating cells and tumorigenesis. Cell.

[98] Tachezy M., Zander H., Wolters-Eisfeld G., Müller J., Wicklein D., Gebauer F., Izbicki J.R., Bockhorn M. (2014). Activated leukocyte cell adhesion molecule (CD166): An “inert” cancer stem cell marker for non-small cell lung cancer?. Stem Cells.

[99] Wang J., Li Z., White J., Zhang L. (2014). Lung Cancer Stem Cells and Implications for Future Therapeutics. Cell Biochem. Biophys..

[100] Bertolini G., Roz L., Perego P., Tortoreto M., Fontanella E., Gatti L., Pratesi G., Fabbri A., Andriani F., Tinelli S. (2009). Highly tumorigenic lung cancer CD133+ cells display stem-like features and are spared by cisplatin treatment. Proc. Natl. Acad. Sci. USA.

[101] Eramo A., Lotti F., Sette G., Pilozzi E., Biffoni M., Di Virgilio A., Conticello C., Ruco L., Peschle C., De Maria R. (2008). Identification and expansion of the tumorigenic lung cancer stem cell population. Cell Death Differ..

[102] Zhang Y., Xu W., Guo H., Zhang Y., He Y., Lee S.H., Song X., Li X., Guo Y., Zhao Y. (2017). NOTCH1 signaling regulates self-renewal and platinum chemoresistance of cancer stem-like cells in human non-small cell lung cancer. Cancer Res..

[103] Meng X., Wang X., Wang Y. (2009). More than 45% of A549 and H446 cells are cancer initiating cells: Evidence from cloning and tumorigenic analyses. Oncol. Rep..

[104] Alama A., Gangemi R., Ferrini S., Barisione G., Orengo A.M., Truini M., Bello M.G.D., Grossi F. (2015). CD133-Positive Cells from Non-Small Cell Lung Cancer Show Distinct Sensitivity to Cisplatin and Afatinib. Arch. Immunol. Ther. Exp..

[105] Watkins D.N., Berman D.M., Burkholder S.G., Wang B., Beachy P.A., Baylin S.B. (2003). Hedgehog signalling within airway epithelial progenitors and in small-cell lung cancer. Nature.

[106] Po A., Silvano M., Miele E., Capalbo C., Eramo A., Salvati V., Todaro M., Besharat Z.M., Catanzaro G., Cucchi D. (2017). Noncanonical GLI1 signaling promotes stemness features and in vivo growth in lung adenocarcinoma. Oncogene.

[107] Li H., Yue D., Jin J.Q., Woodard G.A., Tolani B., Luh T.M., Giroux-Leprieur E., Mo M., Chen Z., Che J. (2016). Gli promotes epithelial-mesenchymal transition in human lung adenocarcinomas. Oncotarget.

[108] Della Corte C.M., Malapelle U., Vigliar E., Pepe F., Troncone G., Ciaramella V., Troiani T., Martinelli E., Belli V., Ciardiello F. (2017). Efficacy of continuous EGFR-inhibition and role of Hedgehog in EGFR acquired resistance in human lung cancer cells with activating mutation of EGFR. Oncotarget.

[109] Bora-Singhal N., Perumal D., Nguyen J., Chellappan S. (2015). Gli1-Mediated Regulation of Sox2 Facilitates Self-Renewal of Stem-Like Cells and Confers Resistance to EGFR Inhibitors in Non-Small Cell Lung Cancer. Neoplasia.

[110] Schnidar H., Eberl M., Klingler S., Mangelberger D., Kasper M., Hauser-Kronberger C., Regl G., Kroismayr R., Moriggl R., Sibilia M. (2009). Epidermal Growth Factor Receptor Signaling Synergizes with Hedgehog/GLI in Oncogenic Transformation via Activation of the MEK/ERK/JUN Pathway. Cancer Res..

[111] Lauth M., Toftgård R. (2007). Non-canonical activation of GLI transcription factors: Implications for targeted anti-cancer therapy. Cell Cycle.

[112] Rosell R., Moran T., Queralt C., Porta R., Cardenal F., Camps C., Majem M., Lopez-Vivanco G., Isla D., Provencio M. (2009). Screening of epidermal growth factor receptor mutation in lung cancer. N. Engl. J. Med..

[113] Teocharoen R., Ruangritchankul K., Vinayanuwattikun C., Sriuranpong V., Sitthideatphaiboon P. (2021). Vimentin expression status is a potential biomarker for brain metastasis development in EGFR-mutant NSCLC patients. Transl. Lung Cancer Res..

[114] Richardson F., Young G.D., Sennello R., Wolf J., Argast G.M., Mercado P., Davies A., Epstein D.M., Wacker B. (2012). The Evaluation of E-Cadherin and Vimentin as Biomarkers of Clinical Outcomes Among Patients with Non-small Cell Lung Cancer Treated with Erlotinib as Second- or Third-line Therapy. Anticancer Res..

[115] Wen G.-M., Mou F.-F., Hou W., Wang D., Xia P. (2019). Integrative Analysis of CD133 mRNA in Human Cancers Based on Data Mining. Stem Cell Rev. Rep..

[116] Janikova M., Skarda J., Dziechciarkova M., Radova L., Chmelova J., Krejci V., Sedlakova E., Zapletalova J., Langova K., Klein J. (2010). Identification of CD133+/Nestin+ Putative Cancer Stem Cells in Non-Small Cell Lung Cancer. Biomed. Pap. Med. Fac. Univ. Palacky Olomouc Czech Repub..

[117] Herpel E., Jensen K., Muley T., Warth A., Schnabel P.A., Meister M., Herth F.J.F., Dienemann H., Thomas M., Gottschling S. (2011). The cancer stem cell antigens CD133, BCRP1/ABCG2 and CD117/c-KIT are not associated with prognosis in resected early-stage non-small cell lung cancer. Anticancer Res..

[118] Okudela K., Woo T., Mitsui H., Tajiri M., Masuda M., Ohashi K. (2012). Expression of the potential cancer stem cell markers, CD133, CD44, ALDH1, and β-catenin, in primary lung adenocarcinoma-their prognostic significance. Pathol. Int..

[119] Mizugaki H., Sakakibara-Konishi J., Kikuchi J., Moriya J., Hatanaka K.C., Kikuchi E., Kinoshita I., Oizumi S., Dosaka-Akita H., Matsuno Y. (2014). CD133 expression: A potential prognostic marker for non-small cell lung cancers. Int. J. Clin. Oncol..

