# Supplementary material for: Polycaprolactone Electrospun Scaffolds Produce an Enrichment of Lung Cancer Stem Cells in Sensitive and Resistant EGFRm Lung Adenocarcinoma
Source: Cancers (Basel). 2021 Oct 22;13(21):5320. doi: 10.3390/cancers13215320 (PMC8582538; doi:10.3390/cancers13215320)
Supplement: Supplementary file 1 [file cancers-13-05320-s001.zip › figureS1.pdf]

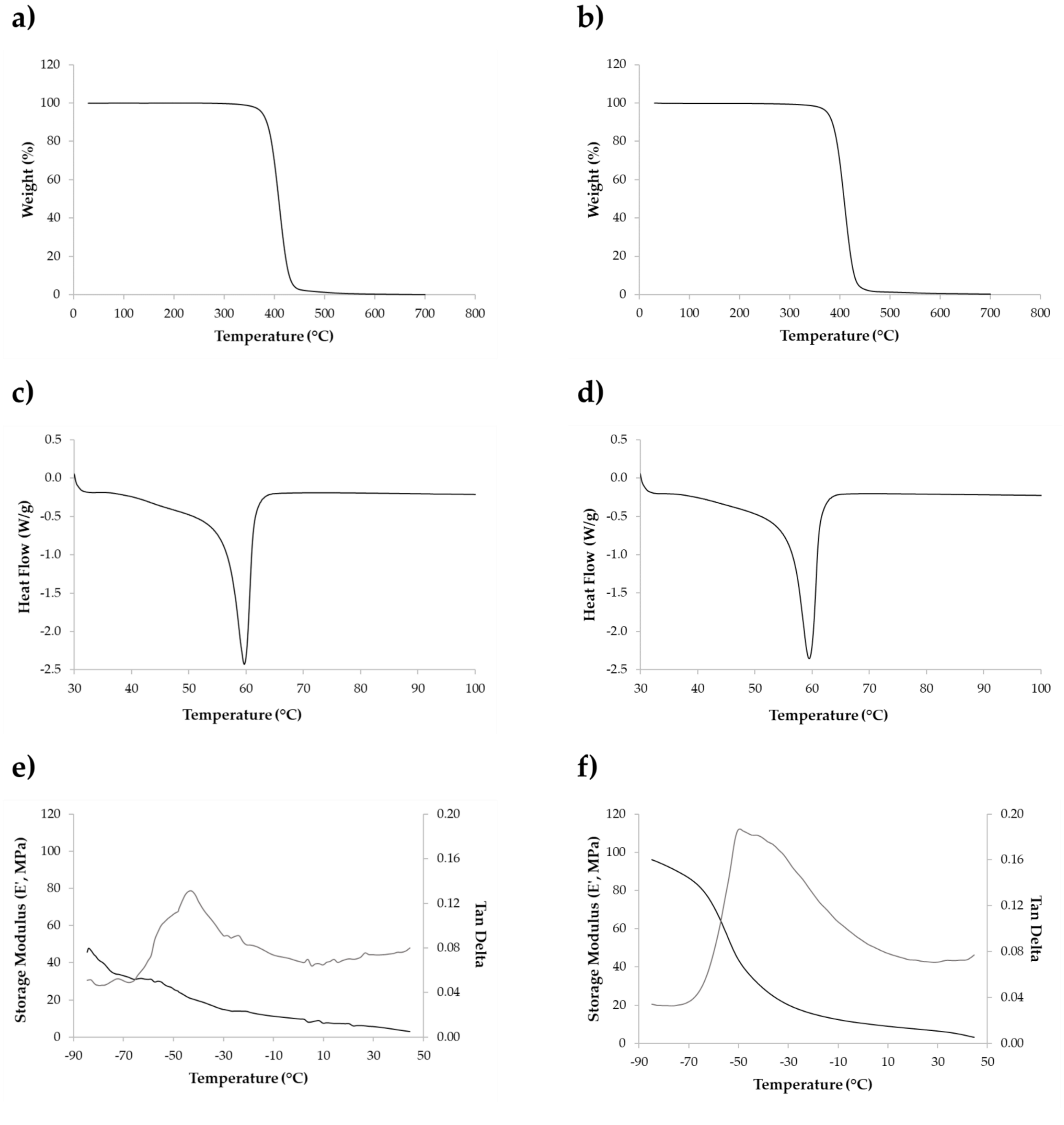

Figure S1. Thermogravimetric analysis of **(a)** 10%-PCL-ES scaffolds and **(b)** 15%-PCL-ES scaffolds. Differential scanning calorimetry of **(c)** 10%-PCL-ES scaffolds and **(d)** 15%-PCL-ES scaffolds. Dynamic mechanical analysis of **(e)** 10%-PCL-ES scaffolds and **(f)** 15%-PCL-ES scaffolds.
