# Supplementary material for: Polycaprolactone Electrospun Scaffolds Produce an Enrichment of Lung Cancer Stem Cells in Sensitive and Resistant EGFRm Lung Adenocarcinoma
Source: Cancers (Basel). 2021 Oct 22;13(21):5320. doi: 10.3390/cancers13215320 (PMC8582538; doi:10.3390/cancers13215320)
Supplement: Supplementary file 1 [file cancers-13-05320-s001.zip › figuresS3.pdf]

PC9

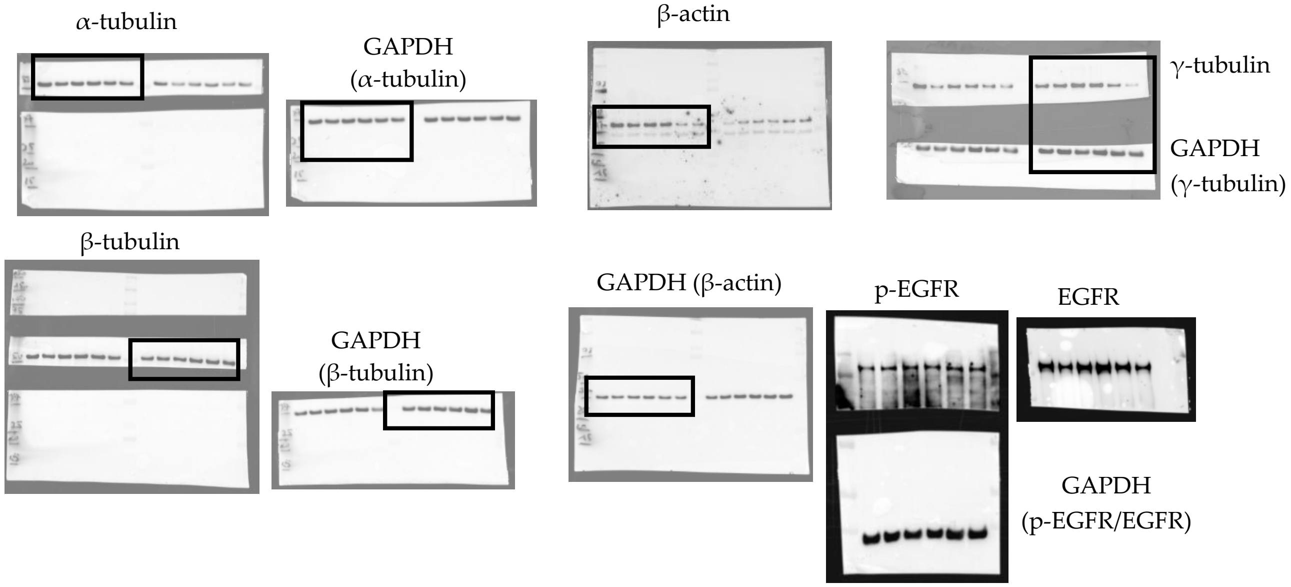

PC9-GR3

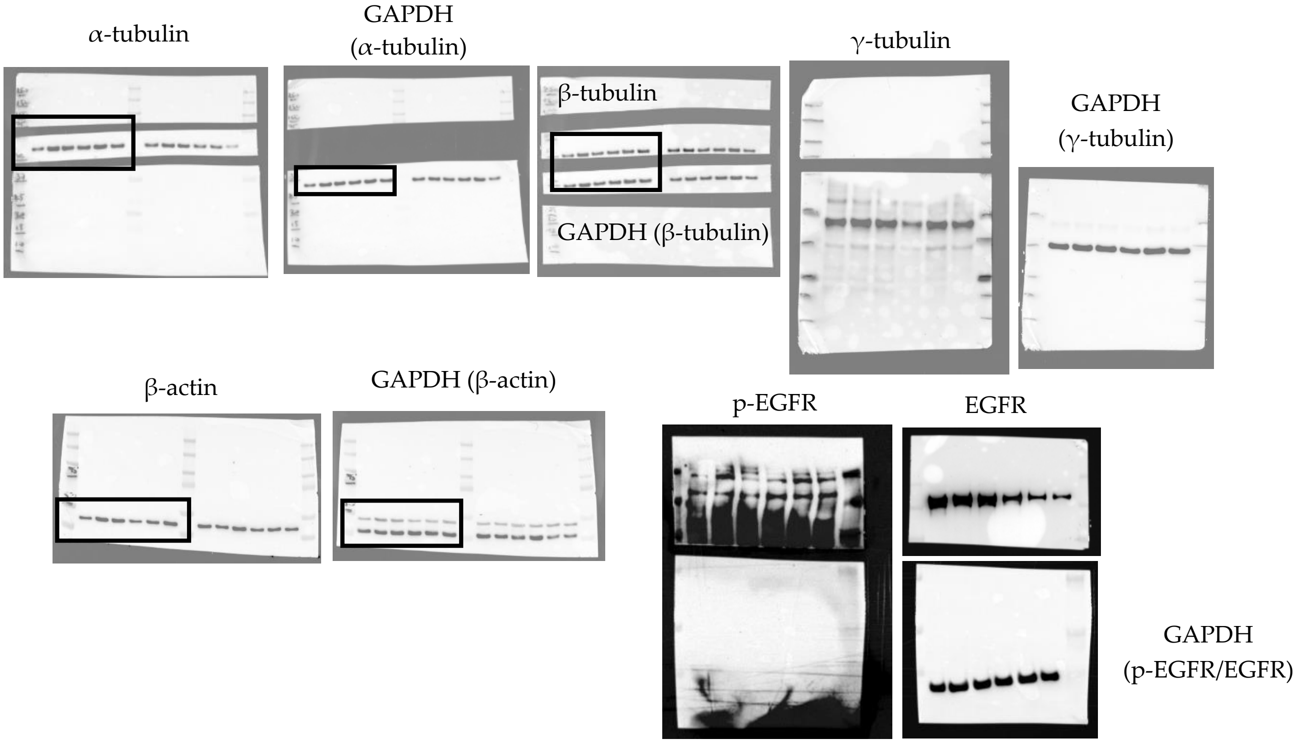

Figure S3. Whole Western blot figures from Figure 3b and Figure 5 showing α-tubulin, β-tubulin, γ-tubulin, β-actin, p-EGFR, EGFR, and GAPDH protein bands with molecular weight markers (merge of colorimetric and chemiluminescence) of PC9 and PC9-GR3.
