# Supplementary material for: Polycaprolactone Electrospun Scaffolds Produce an Enrichment of Lung Cancer Stem Cells in Sensitive and Resistant EGFRm Lung Adenocarcinoma
Source: Cancers (Basel). 2021 Oct 22;13(21):5320. doi: 10.3390/cancers13215320 (PMC8582538; doi:10.3390/cancers13215320)
Supplement: Supplementary file 1 [file cancers-13-05320-s001.zip › figuresS5.pdf]

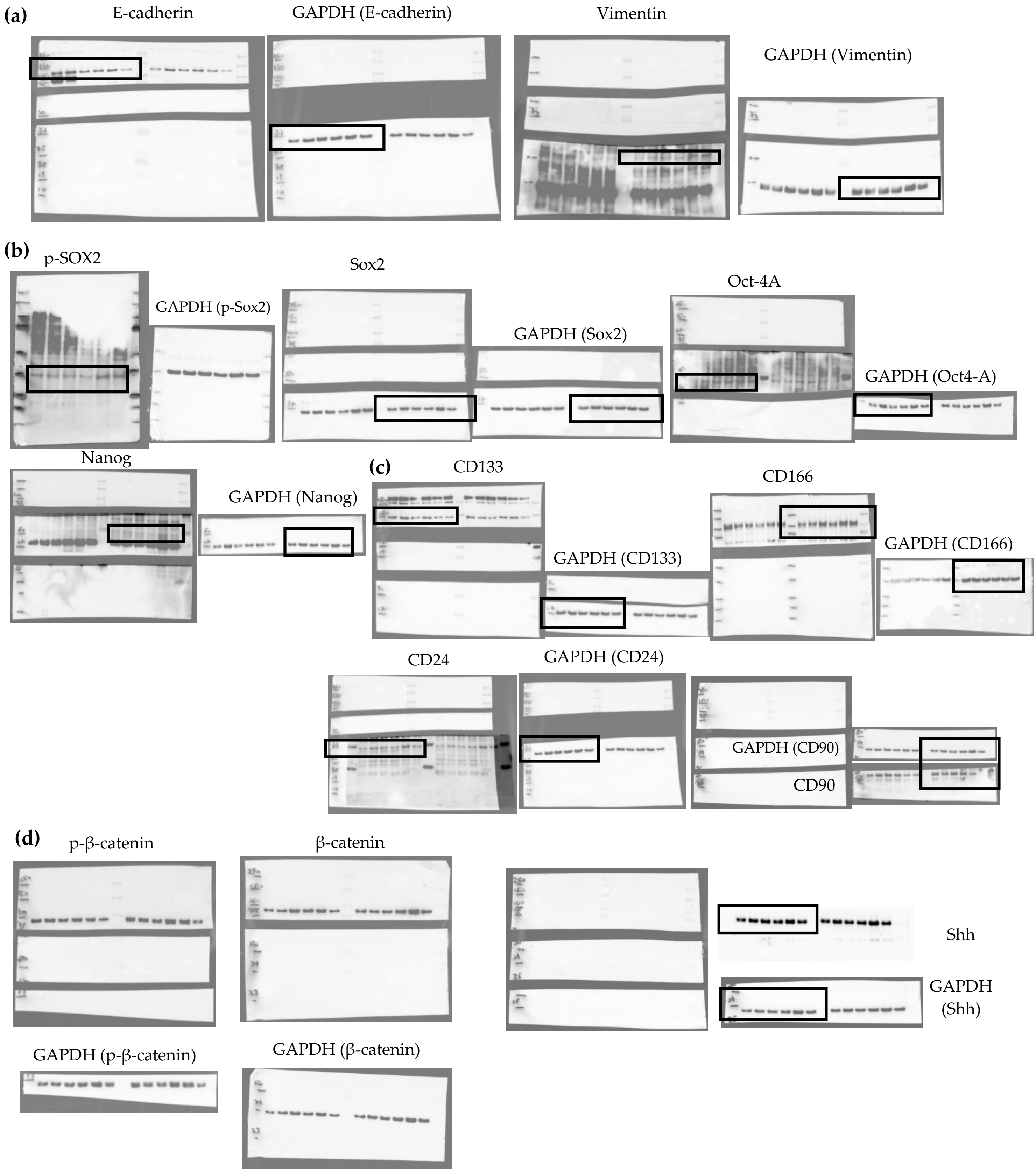

Figure S5. Whole Western blot figures protein bands of **(a)** Figure 6b, **(b)** Figure 7b, **(c)** Figure 8b, and **(d)** Figure 9b with molecular weight markers (merge of colorimetric and chemiluminescence) of PC9-GR3.
