# Supplementary material for: Polycaprolactone Electrospun Scaffolds Produce an Enrichment of Lung Cancer Stem Cells in Sensitive and Resistant EGFRm Lung Adenocarcinoma
Source: Cancers (Basel). 2021 Oct 22;13(21):5320. doi: 10.3390/cancers13215320 (PMC8582538; doi:10.3390/cancers13215320)
Supplement: Supplementary file 1 [file cancers-13-05320-s001.zip › tableS1.pdf]

Table S1. Primer design.

Gene	Reverse sequence (5′-3′)	Forward sequence (5′-3′)
ACTB	CGTGGATGCCACAGGACT	ATTGGCAATGAGCGGTTC
TUBB	GCCCTGGGCACATATTTCT	AGGCTACGTGGGAGACTCG
EGFR	GGGACAGCTTGGATCACACT	CATGTCGATGGACTTCCAGA
ABCB1	CATTTCCTGCTGTCTGCATTGTG	GTCGGACCACCATTGTGATAG
ABCG2	TCGTCCCTGCTTAGACATCC	TGGCTTAGACTCAAGCACAGC
CDH1	CGCTCTCCTCCGAAGAAAC	TGGAGGAATTCTTGCTTTGC
VIM	CATTTCACGCATCTGGCGTTC	AGTCCACTGAGTACCGGAGAC
SNAIL	ATCTCCGGAGGTGGGATG	GCTGCAGGACTCTAATCCAGA
SLUG	GCAGTGAGGGCAAGAAAAAG	GCGATGCCCAGTCTAGAAAA
TWIST	CCTTCTCTGGAAACAATGACATC	AGCTACGCCTTCTCGGTCT
ZEB1	TTTGGCTGGATCACTTTCAAG	GCCAATAAGCAAACGATTCTG
SOX2	GCTTAGCCTCGTCGATGAAC	AACCCCAAGATGCACAACTC
OCT3/4	GGCAGAGGTCGTTTGGCTGAATAGACC	TGGAGAAGGAGAAGCTGGAGCAAAA
NANOG	TGCGTCACACCATTGCTATTCTTC	AATACCTCAGCCTCCAGCAGATG
CD133	GCTTTTCCTATGCCAAACCA	GCCACCGCTCTAGAACTGC
CD166	CATCGTCGTACTGCACACTTT	ACTTGACGTACCTCAGAATCTCA
CD24	AGTGAGACCACGAAGAGACT	CCTCCCAGAGTACTTCCAACT
CD90	CAGGCTGAACTCGTACTGGA	CGCTCTCCTGCTAACAGTCTT
β-CATENIN	ATGGTGCGTACAATGGCAGA	ACTTGCCACACGTGCAATTC
GLI1	CAGAGGTTGGGAGGTAAGGA	CTGGATCGGATAGGTGGTCT
GLI2	CATTGGAGAAACAGGATTGG	GCCCTTCCTGAAAAGAAGAC
PTCH1	GAAGGAGATTATCCCCCTGA	TGACCTAGTCAGGCTGGAAG
PTCH2	CCCAGGACTTCCCATAGAGT	AGGAGCTGCATTACACCAAG
SHH	CAAAGCGTTCAACTTGTCCTTA	GTGGCCGAGAAGACCCTA
GAPDH	CAGAGATGATGACCCTTTTG	TCTTCCAGGAGCGAGATC
