# Supplementary material for: Polycaprolactone Electrospun Scaffolds Produce an Enrichment of Lung Cancer Stem Cells in Sensitive and Resistant EGFRm Lung Adenocarcinoma
Source: Cancers (Basel). 2021 Oct 22;13(21):5320. doi: 10.3390/cancers13215320 (PMC8582538; doi:10.3390/cancers13215320)
Supplement: Supplementary file 1 [file cancers-13-05320-s001.zip › tableS2.pdf]

Table S2. List of antibodies

Protein	Code	Dilution
**WB**		
β-actin	CST;#3700	1:2500
α-tubulin	CST; #3873	1:2500
β-tubulin	ProteinTech; #66240	1:2500
γ-tubulin	ProteinTech; 66320	1:1500
p-EGFR^{Tyr1068}	CST;#2234	1:1000
EGFR	CST; #2232	1:1500
E-cadherin	CST; #3195	1:1000
Vimentin	CST; #5741	1:1500
p-Sox2$^{S250/S251}$	CST; #92186	1:1000
Sox2	CST; #23064	1:1000
Oct-4A	CST; #2840	1:1000
Nanog	CST; #4903	1:1000
CD133	CST; #8678	1:1000
CD166	Abcam; #109215	1:1000
CD24	Abcam; #179821	1:1000
CD90	Abcam; #133350	1:1000
p-β-catenin^{S552}	CST; #5651	1:1000
β-catenin	CST; #9582	1:1000
Shh	CST; #2207	1:1000
GAPDH	CST; #5174	1:2500
**IHC**		
CD133	CST; # 86781	1:700
Vimentin V9	Roche Diagnostics	1:1
