# Supplementary material for: Polycaprolactone Electrospun Scaffolds Produce an Enrichment of Lung Cancer Stem Cells in Sensitive and Resistant EGFRm Lung Adenocarcinoma
Source: Cancers (Basel). 2021 Oct 22;13(21):5320. doi: 10.3390/cancers13215320 (PMC8582538; doi:10.3390/cancers13215320)
Supplement: Supplementary file 1 [file cancers-13-05320-s001.zip › tableS3.pdf]

Table S3. Patient and tumor characteristics at baseline.

	Number	Percentage
**Gender**		
Female	34	75.6
Male	11	24.4
**Age (Years)**		
Median (Q1;Q3)	68	58;74
Mix; Max	46;88	
**Smoking Habit**		
Never	33	73.3
Former	8	17.8
Current	4	8.9
**ECOG**		
0	15	33.3
1	26	57.8
2	4	8.9
**Histology**		
Adenocarcinoma	41	91.2
Adenosquamous	2	4.4
Squamous	2	4.4
**Tumor Differentiation**		
Well	1	2.2
Moderately	16	35.6
Poorly	22	48.9
Missing	6	13.3
**Brain Metastasis**		
No	9	20.0
Yes	35	77.8
Missing	1	2.2
**EGFR Mutation**		
Exon 19	26	57.8
Exon 21	19	42.2
**Response Rate to TKI**		
Complete Response	1	2.2
Partial Response	30	66.7
Stable Disease	7	15.6
Progression Disease	6	13.3
Missing	1	2.2
**CD133 Expression**		
Negative	18	40
Positive	18	40
Missing	9	20
**Vimentin Expression**		
<10%	15	33.3
>10%	21	46.7
Missing	9	20

.
