# Supplementary figures and images for: Polycaprolactone Electrospun Scaffolds Produce an Enrichment of Lung Cancer Stem Cells in Sensitive and Resistant EGFRm Lung Adenocarcinoma
Source: Cancers (Basel). 2021 Oct 22;13(21):5320. doi: 10.3390/cancers13215320 (PMC8582538; doi:10.3390/cancers13215320)

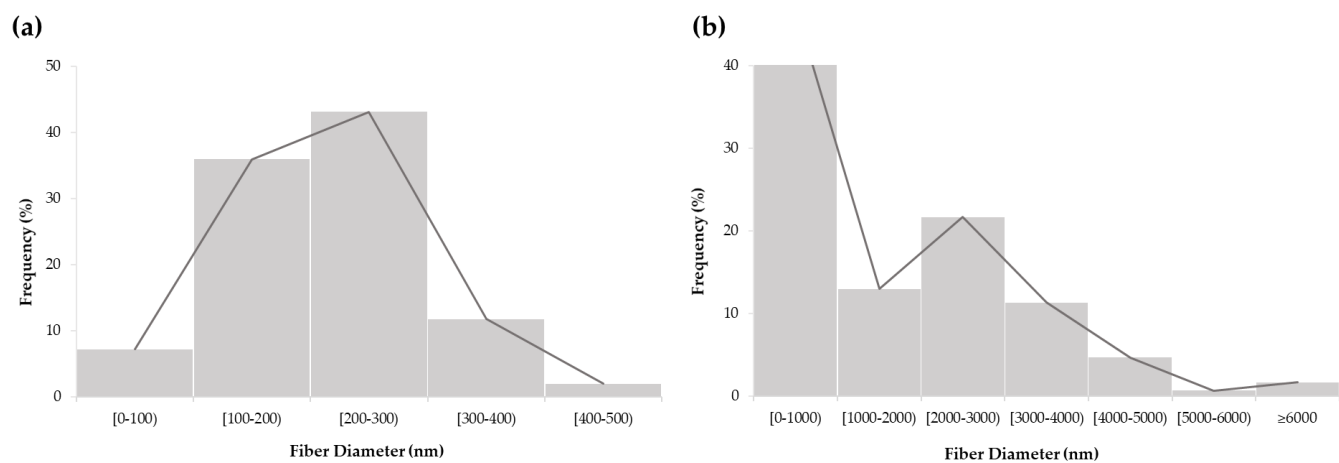

**Figure S2.** Filament diameter histogram of (a) 10%-PCL-ES scaffolds and (b) 15%-PCL-ES scaffolds.

Supplement: Supplementary file 1 [file cancers-13-05320-s001.zip › figureS2.pdf]
